# Systematic Review and Meta-Analysis: Epidemiology of Human *Blastocystis* spp. Infection in Malaysia

**DOI:** 10.3390/tropicalmed8080415

**Published:** 2023-08-15

**Authors:** Vinoth Kumarasamy, Arutchelvan Rajamanikam, Deepa Anbazhagan, Wahib Mohammed Atroosh, Meram Azzani, Vetriselvan Subramaniyan, Syamsa Rizal Abdullah

**Affiliations:** 1 Department of Parasitology & Medical Entomology, Faculty of Medicine, Universiti Kebangsaan Malaysia, Jalan Yaacob Latif, Cheras, Kuala Lumpur 56000, Malaysia; vinoth@ukm.edu.my; 2 Department of Parasitology, Faculty of Medicine, University of Malaya, Kuala Lumpur 50603, Malaysia; 3 Department of Medical Microbiology, International Medical School (IMS), Management & Science University (MSU), Shah Alam 40100, Selangor, Malaysia; 4 Department of Public Health Medicine, Faculty of Medicine, Universiti Teknologi MARA, Sungai Buloh 47000, Selangor, Malaysia; 5 Pharmacology Unit, Jeffrey Cheah School of Medicine and Health Sciences, Monash University, Jalan Lagoon Selatan, Bandar Sunway 47500, Selangor, Malaysia; 6 Center for Transdisciplinary Research, Department of Pharmacology, Saveetha Dental College, Saveetha Institute of Medical and Technical Sciences, Saveetha University, Chennai 600077, Tamil Nadu, India

**Keywords:** *Blastocystis* spp., meta-analysis, subtype distribution, Malaysia

## Abstract

*Blastocystis* spp. is a unicellular enteric protozoan parasite in humans with a controversial role in disease etiology. It is common in developing countries among immunocompromised patients and people who have close contact with animals. In this study, we have systematically reviewed previous studies on the distribution and genotypes of human *Blastocystis* infection in Peninsular Malaysia. Studies examining the prevalence of *Blastocystis* in diverse demographics, including rural, urban, comorbid conditions, and high-risk populations, were taken into consideration. The infection has been reported in nine states; the total percentage of infection was 17.8% (1671/9397), with the most cases in Pahang (27.3%) and the least in Johor (3.4%). Molecular studies revealed the presence of six subtypes: ST1, ST2, ST3, ST4, ST5, and ST6. ST3 was reported as the predominant subtype in all the states, with a prevalence of 54.7% (338/618). The findings provide greater clarity on the epidemiology of *Blastocystis* in Malaysia, which will help in policy making towards planning and strategizing control measures against the parasite.

## 1. Introduction

Intestinal parasite–related illnesses constitute one of the key global public health issues [1,2]. Children from underdeveloped nations and indigenous communities frequently contract these diseases [3]. Often, environmental contamination with human feces is associated with the transmission of these microorganisms [4]. 

*Blastocystis* spp. (*Blastocystis*) is a polymorphic organism having several morphological forms [5,6]. The primary forms of *Blastocystis* are the amoeboid, vacuolar, granular, and cyst [5]. Humans usually contract *Blastocystis* through the fecal–oral route [5]. Person-to-person and zoonotic transmission are common routes of *Blastocystis* transmission [7]. *Blastocystis* divides by binary fission and colonises the large intestine [8]. When attached to the intestinal mucosal layer, *Blastocystis* can produce cysteine proteases that help with pathogenesis [9]. Human-associated *Blastocystis* infection was classified as *Blastocystis hominis*, according to the traditional classification [8]. However, it is already evident that there is cryptic host specificity and large genetic diversity among human *Blastocystis*, and currently DNA sequencing has revealed this organism to be a stramenopile [10]. Recently, the prevalence of *Blastocystis* has been extensively studied in various parts of the world, revealing the various host populations and geographic distribution of this intestinal protozoan parasite [11,12].

*Blastocystis* was initially thought to be a harmless protozoan, but it gained significance as a potential diarrheal pathogen following various reports of its prevalence [13,14]. Although the possible pathogenic mechanism of *Blastocystis* is not fully understood, it remains one of the most frequently identified enteric parasites in humans in developed and developing countries [15,16]. Epidemiological studies globally have demonstrated the distribution of these microorganisms in both healthy and immunocompromised individuals, which suggests low host specificity and has fueled the controversy on the pathogenic nature of this parasite [17]. *Blastocystis* has been associated with various gastrointestinal (GI) symptoms, especially in immunocompromised hosts, exhibiting a characteristic opportunistic pathogenesis [18]. However, *Blastocystis* infections have also been associated with a variety of GI symptoms in immunocompetent individuals, including diarrhea, nausea, vomiting, and flatulence [19,20]. 

Studies have reported increased genetic diversity of *Blastocystis* in humans and animals [21,22,23]. Recent findings based on 16S rRNA have grouped this organism into 28 subtypes (STs) [24]. ST1, ST2, ST3, and ST4 are frequently found in humans [25,26]. ST1 and ST3 are known to infect humans globally [27,28,29] and are known as the most pathogenic genotypes [30,31]. In several rural villages, ST3 was the most prevalent subtype [32,33]. Previous reports have demonstrated that the prevalence of ST3 is very common among children [34,35]. Even though numerous investigations have been carried out, it is still unclear if a specific subtype is related to a particular clinical characteristic. According to previous studies, ST1 and ST3 can infect a wide range of domestic animal species, suggesting that they may spread via zoonotic transmission [36,37].

The many studies of the prevalence of *Blastocystis* infection in Malaysia have indicated that it is an epidemiologically significant intestinal protozoan in the country. People of various racial backgrounds and religions live in Malaysia. Three major races—the Malays, Chinese, and Indians—make up the majority of Malaysians. However, ethnicity has little impact on the prevalence of intestinal parasitic infections in Malaysia. The prevalence of intestinal parasitic infections in Malaysia is more significantly associated with living conditions [38]. Besides *Blastocystis*, other protozoan parasites such as *Entamoeba histolytica*, *Cryptosporidium parvum*, *Isospora belli*, *Cyclospora cayetanensis*, and *Giardia duodenalis* are also commonly identified in the Malaysian population [14]. However, immunosuppressed patients have been known to frequently contract these infections [39].

Consolidated data on the prevalence of *Blastocystis*, its subtypes, and its association with diseases in various groups in Malaysia are not currently available. The collated information would provide a better understanding of the epidemiology of *Blastocystis* in Malaysia and help in informed decision making for implementing and evaluating control strategies. Therefore, we conducted a systematic review and meta-analysis of the available data to assess the prevalence and subtype distribution of *Blastocystis* infections in Malaysia. 

## 2. Materials and Methods

### 2.1. Search Strategy

This review was conducted according to the Preferred reporting items for systematic reviews and meta-analyses (PRISMA) [40]. In November 2021, we searched the relevant articles from the CINAHL, PubMed, Science Direct, and Scopus databases. A manual search of the reference lists of the included articles was also performed to retrieve additional papers that met the eligibility criteria. All the search keywords were identified using the authors’ knowledge and the keywords of published articles on the topic. The keywords used were: *Blastocystis* infection, epidemiology, Malaysia, and their terms and synonyms. There were no restrictions on language or on publication year.

### 2.2. Inclusion Criteria

Original published studies that reported the prevalence of *Blastocystis* among the population in Malaysia and were published before 11 November 2021 were included. There was no restriction on the type of participants or the study setting.

### 2.3. Exclusion Criteria

Articles that reported only the detection of *Blastocystis* without specific findings on epidemiology or prevalence were excluded, as were conference proceedings, case reports, animal studies, and review papers.

### 2.4. Data Extraction

The data extracted from the included articles comprised authors and year of publication, sampling, setting, the method used to detect *Blastocystis*, primary results, conclusion, and quality scoring (Table S1).

### 2.5. Identification

**Figure 1:**
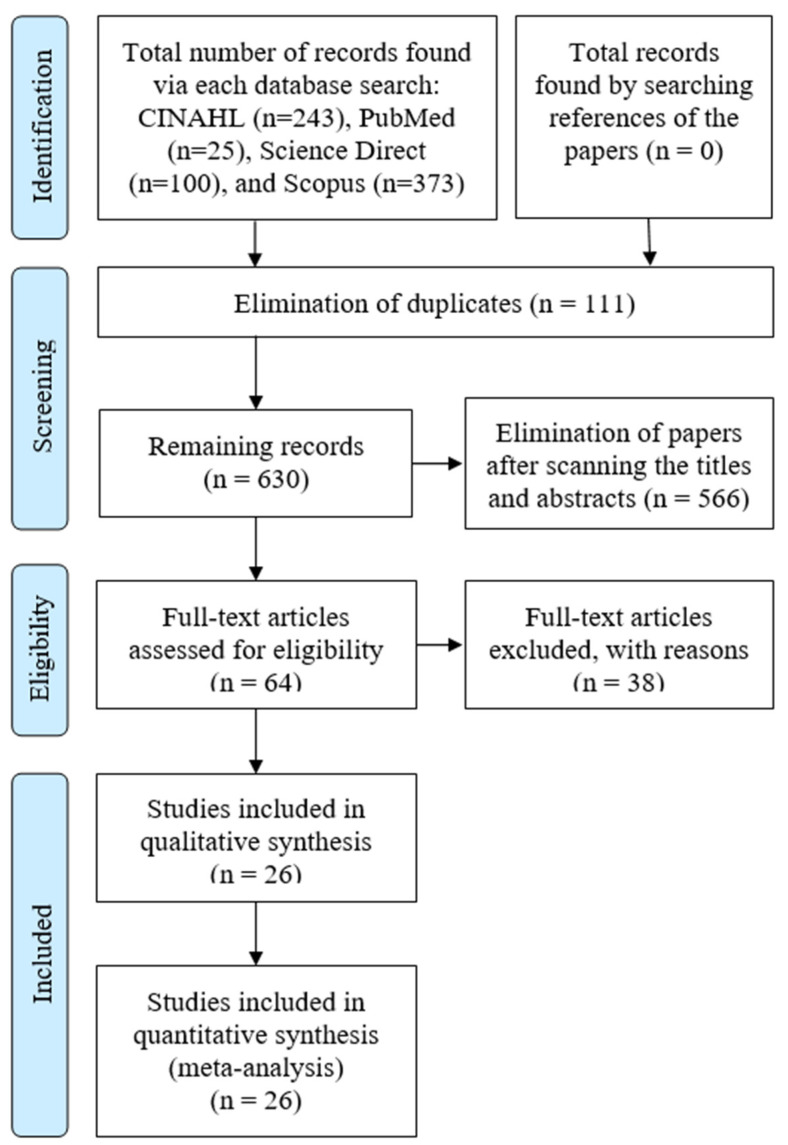
PRISMA flow chart of the included studies.

Figure 1 shows the flowchart of selecting the included papers as agreed upon by all the authors. First, two authors created a search strategy using various keywords and their synonyms. All the search results were moved to the EndNote software (version X9.3.3) (741), and duplicate papers were eliminated (111). Two independent authors screened the titles and abstracts of the remaining 630 papers. Subsequently, a total of 64 papers were selected for full-text analysis. Another two authors assessed the eligibility of the retained papers. Bibliographies of all the papers were searched for pertinent works that fit the eligibility requirements for systematic review but had not been found by searching the various research databases. After thoroughly reviewing the 64 papers, a final 26 papers were selected [15,19,41,42,43,44,45,46,47,48,49,50,51,52,53,54,55,56,57,58,59,60,61,62,63].

The meta-analysis random-effect model was applied to analyze the pooled prevalence, with a 95% confidence interval (CI) of *Blastocystis* infection. The quality of all the included papers was assessed using the BSA Medical Sociology Group survey-based studies. This tool consists of seven questions. Every question answered with a “yes” was given one point, and every question answered with a “no” was given zero points. A score of 1 or 2 was considered low-quality, 3–5 moderate quality, and 6–7 good quality. However, only moderate and high-quality papers were included in the analysis (Table S1).

## 3. Results

The initial study of *Blastocystis* infection in Malaysia was reported in rural areas among the Malay and Orang Asli (indigenous people of Malaysia) ethnic groups [41]. Subsequently, studies were conducted throughout Malaysia, mostly among school children and animal handlers, between 1992 and 1999 [41,52,57,59]. The 26 studies included in this review evaluated the prevalence of *Blastocystis* among school children and adults from rural and urban areas; patients with comorbidities (pediatric, cancer, HIV, dengue patients, etc.); and high-risk groups such as prisoners, migrant workers, and animal handlers. A total of 9596 stool samples were collected as seen in the 26 included studies, with an overall *Blastocystis* prevalence of 17.6%. There were 338 samples that tested positive for ST3, 170 for ST1, 62 for ST2, 27 for ST4, 10 for ST5, 1 for ST6, and 10 for mixed ST.

Most of the studies (*n* = 18) investigated the prevalence of parasitic infections among individuals from rural areas [35,38,41,42,43,44,45,46,47,48,49,50,51,52,53,54,55,56]. The prevalence of *Blastocytis* ranged from 1.1% to 83.7% in these areas. Eight of the 26 studies provided molecular characterization of *Blastocystis* subtypes using small subunit ribosomal (SSU rRNA) gene sequencing [15,35,42,44,45,61,62,63]. The remaining 17 studies used direct fecal microscopy as their gold-standard diagnostic method [35,38,41,43,46,47,48,49,50,51,52,53,54,55,56,57,58,59,60]. 

Peninsular Malaysia comprises 11 states, and *Blastocystis* infection has been reported in most of the states. Only one study reported the prevalence of *Blastocystis* across various states in Malaysia [35]. The rest of the studies were conducted in specific locations in different states; ten were performed in Selangor [15,35,41,52,56,57,58,60,61,62,63], seven in Pahang [35,42,43,44,45,46,51,53,54,55], three in Perak [35,47,48,49], and one each in Kelantan [59], Terengganu, [50], and Negeri Sembilan [38].

**Figure 2:**
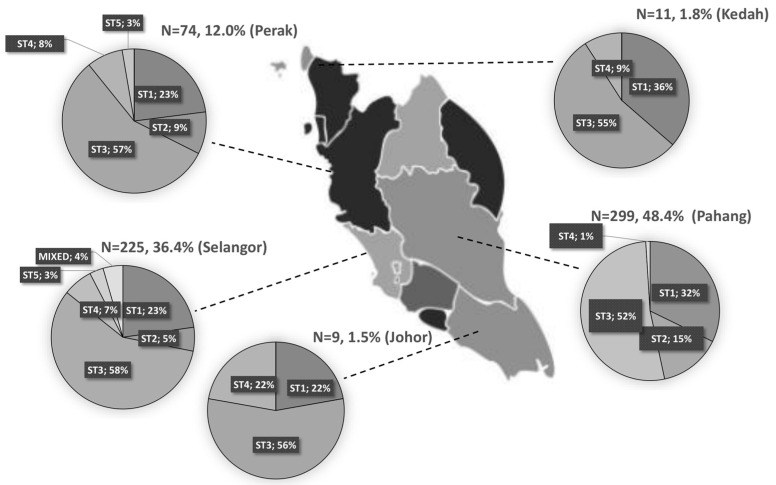
*Blastocystis* subtype distributions across various states in Peninsular Malaysia based on molecular studies.

Since 2009, more Malaysian studies have used PCR-based analysis, making statistics on the prevalence of *Blastocystis* subtypes available [61]. Based on PCR analyses, Pahang showed the highest prevalence of *Blastocystis* (48.4%), followed by Selangor (36.4%) (Figure 2). The sequence analysis of the Malaysian isolate’s SSU rRNA gene barcode region revealed six *Blastocystis* subtypes (Table 1). Most of the samples represented infections with a single subtype (ST1, ST2, ST3, ST4, ST5, or ST6). Among these, ST3 was the most prevalent *Blastocystis* subtype, seen in the most studies in Malaysia (54.7%, 338/618) (Figure 2). ST1 and ST3 were detected in most of the states in Malaysia including Selangor, Pahang, Perak, Johor, and Kedah. The ST6 subtype was the least prevalent (0.2%) and was reported in the state of Selangor among prisoners (Table 1). In the state of Perak and Selangor, the highest number of subtypes were identified, including ST1, ST2, ST3, ST4, and ST5. In the state of Pahang, four different subtypes were found, including ST1, ST2, ST3, and ST4. In the states of Kedah and Johor, three different subtypes were identified, including ST1, ST3, and ST4 (Figure 2).

**Figure 3:**
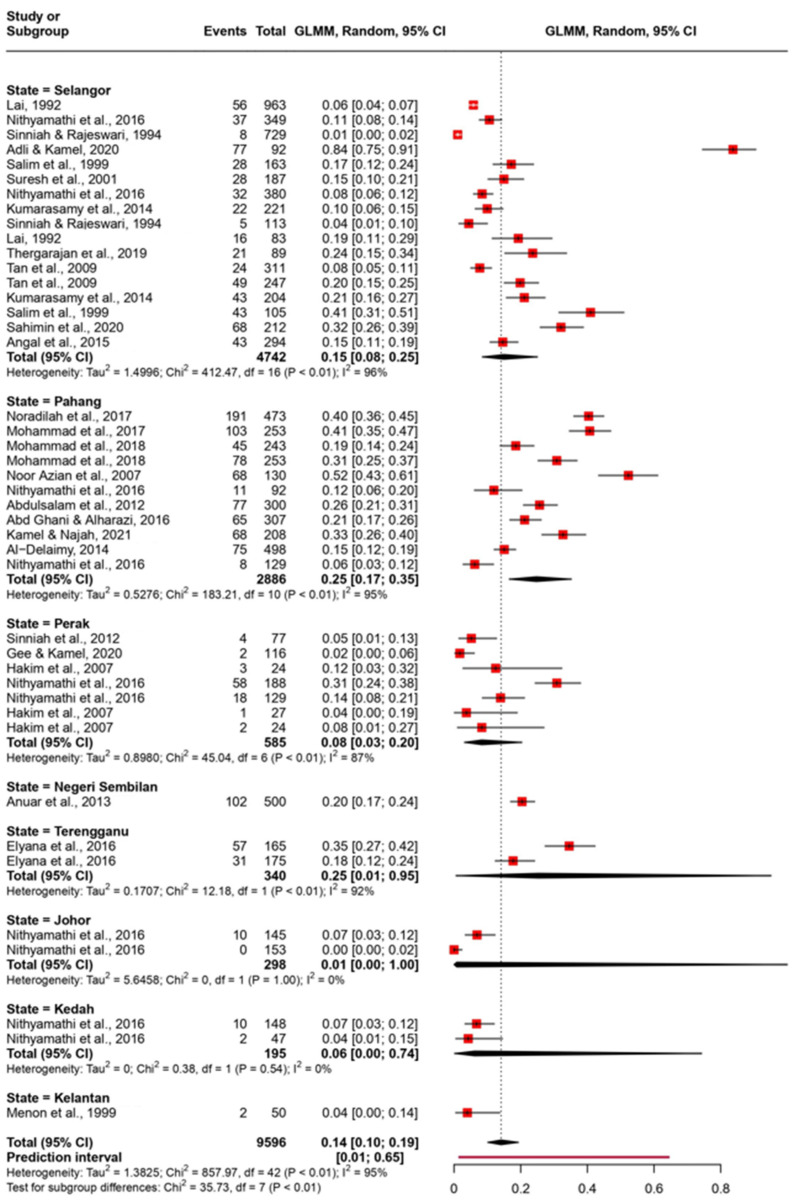
Forest plot of pooled prevalence of *Blastocystis* in 7 states of Peninsular Malaysia using a random-effects model and 95% CI.

**Figure 4:**
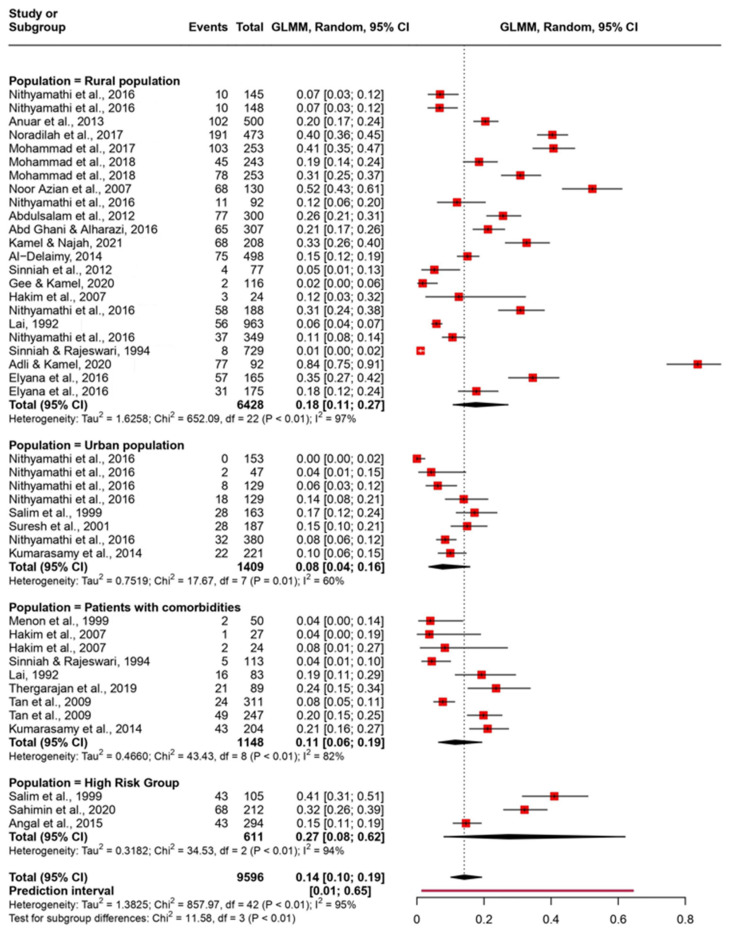
Forest plot of pooled prevalence of *Blastocystis* in various cohorts in Malaysia using a random-effects model and 95% CI.

**Figure 5:**
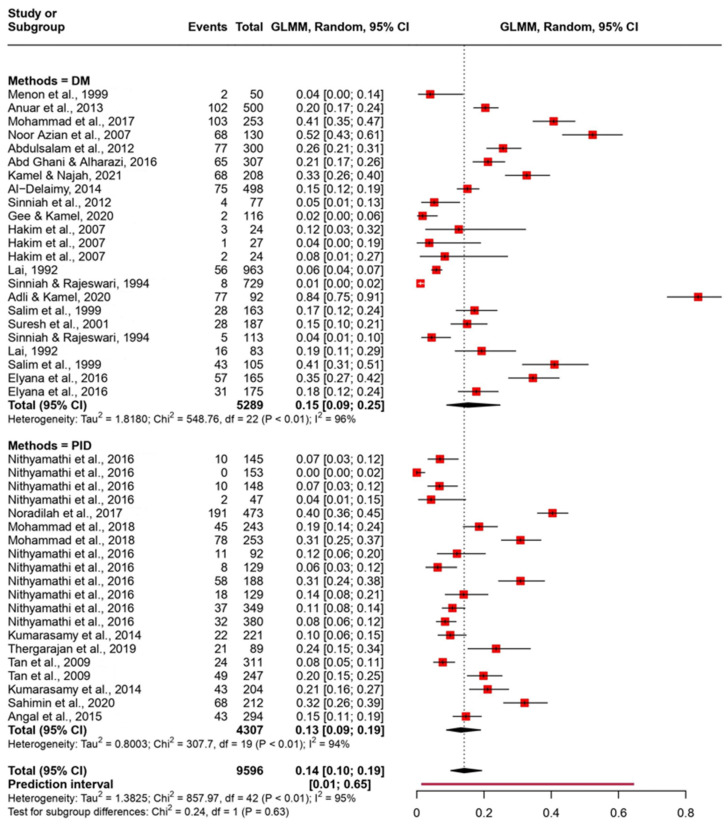
Forest plot of pooled prevalence of *Blastocystis* based on the method of diagnosis in Malaysia using a random-effects model and 95% CI.

The pooled prevalence was evaluated using the random-effect model with the Generalized Linear Mixed Model. The pooled prevalence was evaluated with different groupings on the basis of state, diagnosis method, and type of population (Figure 3, Figure 4, and Figure 5, respectively). We observed that the pooled prevalence was highest in the state of Pahang with about 25% (95% CI: 17–35%) (Figure 3). The pooled prevalence was also notably high in high-risk populations, with about 27% (95% CI: 8–62%) with a wide confidence interval (Figure 4). The method of diagnosis was grouped into direct microscopy (DM) and PCR, in vitro cultivation, and direct stool smear (PID). Both DM and PID demonstrated a similar prevalence of 15% (95% CI: 9–25%) and 13% (95% CI: 9–19%), respectively (Figure 5). 

## 4. Discussion

Amidst a myriad of microorganisms inhabiting the gut flora, *Blastocystis*, the only stramenopile infecting humans [64], appears to be one of the most common protozoan parasites [65], with colonization reported as being as high as 100% in some developing countries [66]. Nine out of the at least 17 subgroups of this parasite that have been described by phylogenetic analysis were found in humans [11]. The ST3 isolate is responsible for the most human infections globally [27,67,68]. Numerous cases of *Blastocystis* infection have been documented in several Asian nations [69]. Although humans and a few other animal species were the most frequently reported hosts, this parasite have also been found in various water sources [70]. In addition, there have been studies showing foodborne transmission of this parasite [71]. One of the major risk factors for *Blastocystis* carriage appears to be the immunological status of the hosts. Populations with compromised immune systems are more vulnerable to *Blastocystis* carriage [72]. There have been many in vitro and in vivo studies associating *Blastocystis* with pathogenesis. Recently, *Blastocystis* has been associated with the proliferation of colorectal cancer cells in vitro [73], and exacerbation of colon carcinogenesis in infected Wistar rats has been shown [74]. To the contrary, most research on the gut microbiota has shown that *Blastocystis* is a typical component of the healthy gut microbiota, that it is linked to higher bacterial populations, and that chronic asymptomatic infection is typical [75].

Studies on the prevalence of *Blastocystis* in different states or populations in Malaysia have surfaced sporadically over the years. However, a systematic collection of such prevalence data and a statistical conclusion that could contribute to controlling and managing *Blastocystis* infection is unavailable. Thus, the present study has consolidated epidemiological data from different states and cohort groups to present an overview of *Blastocystis* infections and their subtype distributions in Peninsular Malaysia. 

Numerous elements, including the immune health of the host, geographic regions, and host age and dietary habits, may impact the occurrence of *Blastocystis* infection. Moreover, the sensitivity of the screening methods used is another crucial variable that can have an impact on the prevalence rate. Wet mount smears have been used to identify *Blastocystis* as a gold-standard method in the majority of previous studies. This was carried out by looking for vacuolar, granular, amoebic, or cystic forms of the parasite in the stool samples microscopically [76]. However, detecting these parasites via this technique was made more difficult by the irregular shedding feature of *Blastocystis* [77]. Therefore, more advanced techniques such as molecular methods were utilized in recent studies [78,79]. The results obtained from this systematic review showed that some studies used only direct microscopy (DM) as their identification method whereas other studies used three different methods including PCR, in vitro cultivation, and direct stool smear (PID) in their studies. However, the prevalence rate of *Blastocystis* infection was almost similar using DM alone or with a combination of different diagnostic methods. Hence, it can be seen that direct microscopy is just as sensitive as other techniques like PCR. However, the molecular method was useful in various studies to identify genotype distributions.

Most of the prevalence studies included in this systematic review did not report specific gastrointestinal symptoms associated with *Blastocystis*. Two studies that were carried out among pediatric patients with gastrointestinal symptoms showed a low prevalence of *Blastocystis* of about only 4% [52,59]. In another study, both symptomatic and asymptomatic *Blastocystis* infection was reported among children from rural settings and aborigines from the state of Pahang [54]. Infection with *Blastocystis* was found to be significantly associated with gastrointestinal symptoms among these schoolchildren, with abdominal pain and diarrhea being the most common symptoms [46,54]. Similarly, abdominal discomfort was discovered to be the primary symptom in a study conducted in Switzerland that revealed high *Blastocystis* infection in symptomatic children [19]. Among aborigines of mixed ages, more people with *Blastocystis* infection were asymptomatic than symptomatic, and the symptoms primarily included fever and diarrhea [46]. Therefore, the pathogenic role of *Blastocystis* is still debatable [17]. Many clinical and epidemiological studies concluded that *Blastocystis* is a commensal organism and, when found, is probably not the cause of any clinical symptoms [25,80,81]. Whether *Blastocystis* exerts subliminal influence in a host is not known. Yet some studies have indicated an association between certain gastrointestinal symptoms and *Blastocystis* infection [19,20]. Moreover, several studies found a higher incidence of *Blastocystis* infection among immunocompromised individuals, including AIDS patients [18].

The inferred pooled prevalence of *Blastocystis* in the Malaysian population based on the 26 publications (43 datasets) was 14% (95% CI: 10–19%). Due to the unavailability of a previously published meta-analysis of *Blastocytis* infection in Malaysia, a direct comparison of our findings is not possible. Previously, a meta-analysis among the Iranian and Brazilian populations demonstrated a pooled prevalence of 9.1% (95% CI: 8.2–10.1%) in Iran [82] and 24% (95% CI: 22–27%) in Brazil [11]. The different detection methods or the differences in living conditions of the populations were likely the cause for these variations. Direct microscopy, PCR, and/or in vitro cultivation are often used in the detection of *Blastocystis* prevalence; however, in Malaysia, in vitro cultivation is the most common method.

The pooled prevalence based on state was highest in the states of Pahang and Terengganu (25%), followed by Selangor (15%), Perak (8%), Kedah (6%), and Johor (1%). Negeri Sembilan and Kelantan were excluded from the comparison, since only one prevalence study was reported in these states. Moreover, there are no reports on the prevalence of *Blastocystis* in Penang, Perlis, Melaka, Sabah, or Sarawak. Most of the studies were conducted in Selangor, with up to 17 datasets and 4543 samples. We found that the pooled prevalence of this state was 15% (95% CI: 8.0–25.0%). According to the Malaysian Department of Statistics (DOSM), Selangor is the most populous state and is regarded as an economic powerhouse, contributing a large portion of the nation’s gross domestic product. The maintenance of public health in this fast-industrialized state could drive more research interest. Also, the presence of established research institutes and universities in the region drives research activity, thus resulting in more scientific outputs. However, compared to Pahang, the prevalence of *Blastocystis* was lower in Selangor. This could be attributed to the higher socioeconomic development in Selangor, contributing to improved hygiene practices and sanitation. A recent study demonstrated that industrialization contributed to the influx of migration [83]. These researchers noted that the highest rural-to-urban migration rate in Malaysia was in Selangor. Thus, migration may be contributing to Selangor’s high *Blastocystis* prevalence despite its socioeconomic development. According to Rashid [83], Malaysia is still experiencing social landscape changes; thus, a continual increase in migration can be expected. The influx of foreign migrants is rising due to the increasing demand for factory workers in Selangor. These foreigners have been shown to have a high parasitic burden upon arrival in the country [62]. This observation implies the need for adequate support and planning in managing migration, especially involving foreign migrant workers and changes in the internal social landscape.

A previous epidemiological study across Malaysia showed that *Blastocystis* infection is more prevalent in the rural (13.7%) than in the urban population (3.4%) [35]. Similarly, in the present study, the pooled prevalence of *Blastocystis* in rural areas was higher than in the urban areas, i.e.,18.0% (95% CI: 11.0–27.0%) and 7.0% (95% CI: 13.0–21.0%), respectively (Figure 4). In rural areas, a higher *Blastocystis* prevalence was observed among indigenous individuals than Malay villagers. Among Orang Asli school children, a higher prevalence was found in Selangor and Pahang compared to Perak, even though the living conditions in both provinces are similar [47,48,51,53,54,56]. According to earlier studies, the disparity in prevalence is primarily caused by a wide range of risk factors for *Blastocystis* infections. Drinking water was reported to be the main source of *Blastocystis* infection among rural primary school children in Pahang [51]. In the past, it was discovered that the ingestion of unboiled water was associated with a high prevalence of *Blastocystis* infections in other countries [84,85]. In addition to drinking untreated water, the presence of other *Blastocystis*-infected family members was the main reason for the high prevalence of *Blastocystis* among indigenous people in Negeri Sembilan, Perak, and Pahang [38,41].

The high pooled prevalence of *Blastocystis* observed in Pahang and Terengganu may thus be due to their high rural population. In a recent report by DOSM, these states comprised a relatively high percentage of the rural population, i.e., 47.2% in Pahang and 35.8% in Terengganu. However, we also noted an imbalance in the number of reports and sample size based on states. Only one study reported on the urban and rural population in Terengganu by sampling 340 people [50]. In contrast, there were 11 studies with a total of 2886 samples in Pahang. Other states with limited or no reported data on *Blastocystis* infection may contribute to bias in conclusions. The high heterogeneity and the small number of studies also may impact the statistical power of these tests. Thus, more research is needed to determine the prevalence of *Blastocystis* in the various states in Malaysia.

Investigations of the prevalence of *Blastocystis* infections in Malaysia’s various communities have mostly been conducted in Peninsular Malaysia, but little is known about the prevalence and risk factors of the disease among general populations in Sabah and Sarawak. Between 2010 and 2015, the bulk of research on *Blastocystis* was carried out in Peninsular Malaysia and mostly involved school children, aborigines, and rural residents. Fewer studies were carried out among individuals from high-risk groups. High-risk-group individuals such as animal handlers, prisoners, and migrant workers are the most prone to contracting *Blastocystis*, i.e., at 27% (95% CI: 8.0–62.0%). Prisoners and migrant workers primarily exhibited nonspecific gastrointestinal symptoms. Comparable outcomes were seen in food handlers and immigrant laborers who were routinely tested in Iran and Qatar, respectively, for *Blastocystis* [86,87]. Their studies proved that there was a risk of *Blastocystis* infection among those who worked closely with animals. In this systematic review, ten individuals with ST5 infection in a mixed population and one ST6 case in a prisoner were documented [63]. ST5 was found among school children from rural areas (*n* = 3), in a mixed-age population from urban areas (*n* = 6), and in a colorectal cancer patient (*n* = 1). Previously, these subtypes were associated with domestic animals [88,89]. In the past, ST6 was seen among chicken slaughterhouse workers in Lebanon [88], and ST5 was seen among pig handlers in Thailand [89]. Other subtypes, such as ST7–ST14, were also frequently found in domestic animals and wild animals in prior studies conducted in Malaysia and elsewhere [90,91] but were not observed in humans in our systematic review. The presence of ST5 and ST6 among various cohorts in Malaysia indicates zoonotic transmission, as these subtypes have been reported among the domestic animals [92].

The controversial pathogenic nature of *Blastocystis* prevents incriminating it as having a direct causative role in symptomatic cases. According to several studies, this parasite frequently leads to opportunistic infection in immunocompromised patients, producing various gastrointestinal symptoms [61]. Migrant workers and prisoners live in close proximity, often overcrowded, with low sanitation levels; thus, they are continuously exposed to *Blastocystis* infection [62], leading to increased symptomatic infections in these populations. A similar finding was reported previously in Qatar whereby more than 70% newly arriving immigrant workers were detected positive for *Blastocystis* [87]. Similar to Qatar, once a worker in Malaysia receives a work permit, they are not required to go through any additional health inspections, unless they work in the food business, in which case an annual re-examination is required. Therefore, housekeepers and other migrant workers may act as a possible reservoir and point of infection for gastrointestinal infections among locals. 

Our study corroborates other reports on the opportunistic nature of *Blastocystis* infection [93,94]. The pooled prevalence of 11% was seen in individuals who were comorbid with cancer, acquired immunodeficiency syndrome (AIDS), and suffered from chronic diarrhea (Figure 4). Although these individuals were mainly from urbanized areas, the prevalence of *Blastocystis* was greater than in healthy urbanites, who had a pooled prevalence of 7%. Studies have previously reported the ability of *Blastocystis* to influence host immune responses by triggering inflammatory cytokines [95] and degradation of IgA [96]. The current understanding of opportunistic *Blastocystis* infection highlights its occurrence in immunocompromised individuals; however, the direct influence of this organism and the mechanism involved in its role as an opportunistic infection is still not understood.

Recent *Blastocystis* knowledge has evolved to identify subtype level differences [82] and even variations within subtypes [83]. The distribution of *Blastocystis* subtypes was based on reports from Selangor, Perak, Kedah, Pahang, and Johor. It comprises primarily ST3 (more than 50% prevalence), followed by ST1 (27.5%). The absence of subtype information in many studies restricts the complete elucidation of subtype distribution in Malaysia. Nevertheless, our finding parallels another study that showed ST3 as the predominant subtype in the Southeast Asia region [97] and in tropical underdeveloped nations [67,94,98]. Also, a prior study of patients in a Singaporean hospital found that *Blastocystis* ST3 was the most prevalent genotype isolated [99]. Similarly, ST3 was the most identified genotype among aborigines living in the state of Pahang and Perak. In contrast, in a study carried out in Brazil among an indigenous population, ST3 was found to be the least prevalent genotype [100]. According to reports, *Blastocystis* ST3 has a human origin as being part of healthy gut microbiota [25]. Detection of other subtypes such ST1, ST2, ST4, and ST5 in other states, particularly Selangor and Perak, shows the high possibility of zoonotic transmission. These STs have been previously commonly identified in zoo animals in France and Malaysia [101,102]. 

Subtype–symptom association is still unclear, and several studies have attempted to link pathogenicity to a specific *Blastocystis* subtype. A study by Nithyamathi et al. [35] found that ST3 is predominant in symptomatic infection. A more palatable explanation is that not all strains of a subtype are pathogenic and that subtype is not the sole factor influencing pathogenicity [103,104]. A recent study has demonstrated variation due to the source of isolation in a single subtype. This suggests the influence of the microenvironment on *Blastocystis* and its infection [105]. Hence, to understand the dynamics of *Blastocystis* infection and its association with pathogenesis, it is necessary to include subtype information in all future epidemiological studies. 

## 5. Conclusions

Our study has provided a better understanding of the current *Blastocystis* epidemiology in Malaysia. Human infection has been reported in various states in Malaysia, mainly in Selangor, Pahang, and Perak. The overall prevalence is high (17.8%), with reports of up to 25%, especially in patients with comorbidities. There are six distinct subtypes of *Blastocystis* isolated from humans, with ST3 being the most prevalent. Although there is evidence of fecal–oral transmission of *Blastocystis* cysts, the precise means of transmission across the different hosts and/or between animals and people has not yet been proven. More research is needed to assess and understand the pathogenicity, mode of transmission, and host specificity of various *Blastocystis* subtypes. Currently, it is clear that there is a growing interest in studies related to *Blastocystis* and its pathogenesis. This interest is important for understanding the vast amount of information on the epidemiology and pathogenicity of this microorganism, which are as yet little known. 

## Figures and Tables

**Table 1:** Studies on the prevalence of *Blastocystis* in Malaysia.

No.		Year of Study	States	Methods	No. Examined	No. Positive	Prevalence (%)	Gastrointestinal Symptoms	Subtypes							Author/Year
									ST1	ST2	ST3	ST4	ST5	ST6	MIXED	
	**Rural population**		**Rural population**													
1	General population from rural area	1982–1992	Selangor	DM	963	56	5.8	Asymptomatic								Lai, 1992 [41]
2	Aborigines	2014–2015	Pahang	PID	473	191	40.4	Asymptomatic	63	27	98	3	0	0	0	Noradilah et al., 2017 [42]
3	Aborigines	2015	Pahang	DM	253	103	40.7	Asymptomatic								Mohammad et al., 2017 [43]
4	Aborigines	2016	Pahang	PID	243	45	18.5	Asymptomatic	14	7	24	0	0	0	0	Mohammad et al., 2018 [44]
5	Aborigines	2015	Pahang	PID	253	78	30.8	Symptomatic & Asymptomatic	14	7	24	0	0	0	0	Mohammad et al., 2018 [45]
6	Aborigines	2006	Pahang	DM	130	68	52.3	Asymptomatic								Noor Azian et al., 2007 [46]
7	Aborigines	2011	Perak	DM	77	4	5.2	Asymptomatic								Sinniah et al., 2012 [47]
8	Aborigines	2011	Negeri Sembilan	DM	500	102	20.4	Asymptomatic								Anuar et al., 2013 [38]
9	Aborigines	2018	Perak	DM	116	2	1.7	Asymptomatic								Gee & Kamel, 2020 [48]
10	Aborigines	2004	Perak	DM	24	3	12.5	Asymptomatic								Hakim et al., 2007 [49]
11	Aborigines	2014–2015	Terengganu	DM	165	57	34.5	Asymptomatic								Elyana et al., 2016 [50]
12	Malays	2014–2015	Terengganu	DM	175	31	17.7	Asymptomatic								Elyana et al., 2016 [50]
13	Schoolchildren from rural area	2012–2013	Perak	PID	188	58	30.9	Asymptomatic	12	7	32	5	2	0	0	Nithyamathi et al., 2016 [35]
14	Schoolchildren from rural area	2012–2013	Johor	PID	145	10	6.9	Asymptomatic	2	0	5	2	0	0	0	Nithyamathi et al., 2016 [35]
15	Schoolchildren from rural area	2012–2013	Selangor	PID	349	37	10.6	Asymptomatic	10	3	22	1	1	0	0	Nithyamathi et al., 2016 [35]
16	Schoolchildren from rural area	2012–2013	Pahang	PID	92	11	12.1	Asymptomatic	2	1	7	0	0	0	0	Nithyamathi et al., 2016 [35]
17	Schoolchildren from rural area	2012–2013	Kedah	PID	148	10	6.8	Asymptomatic	4	0	4	1	0	0	0	Nithyamathi et al., 2016 [35]
18	Schoolchildren from rural area	2010	Pahang	DM	300	77	25.7	Symptomatic & Asymptomatic								Abdulsalam et al., 2012 [51]
19	Schoolchildren from rural area	1990	Selangor	DM	729	8	1.1	Asymptomatic								Sinniah & Rajeswari, 1994 [52]
20	Aborigine School Children	2008	Pahang	DM	307	65	21.2	Asymptomatic								Abd Ghani & Alharazi, 2016 [53]
21	Aborigine School Children	2014	Pahang	DM	208	68	32.7	Asymptomatic								Kamel & Najah, 2021 [54]
22	Aborigine School Children	2012	Pahang	DM	498	75	15.1	Asymptomatic								Al-Delaimy, 2014 [55]
23	Aborigine School Children	2017	Selangor	DM	92	77	83.7	Asymptomatic								Adli & Kamel, 2020 [56]
	**Urban population**		**Urban population**													
1	General population from flats in city	1998	Selangor	DM	163	28	17	Asymptomatic								Salim et al., 1999 [57]
2	General population from flats in city	1998	Selangor	DM	187	28	14.9	Asymptomatic								Suresh et al., 2001 [58]
3	Schoolchildren from urban area	2012–2013	Perak	PID	129	18	14	Asymptomatic	5	0	10	1	0	0	0	Nithyamathi et al., 2016 [35]
4	Schoolchildren from urban area	2012–2013	Johor	PID	153	0	0	Asymptomatic	0	0	0	0	0	0	0	Nithyamathi et al., 2016 [35]
5	Schoolchildren from urban area	2012–2013	Selangor	PID	380	32	8.2	Asymptomatic	6	2	17	3	2	0	0	Nithyamathi et al., 2016 [35]
6	Schoolchildren from urban area	2012–2013	Pahang	PID	129	8	6.2	Asymptomatic	3	1	3	0	1	0	0	Nithyamathi et al., 2016 [35]
7	Schoolchildren from urban area	2012–2013	Kedah	PID	47	2	4.3	Asymptomatic	0	0	2	0	0	0	0	Nithyamathi et al., 2016 [35]
8	General population from urban area	2010–2012	Selangor	PID	221	22	9.95	Asymptomatic	9	1	26	0	3	0	4	Kumarasamy et al., 2014 [15]
	**Patients with comorbidities**															
1	Children with diarrhea	1990	Selangor	DM	113	5	4.4	Diarrhea								Sinniah & Rajeswari, 1994 [52]
2	Patients with chronic diarrhea	1982–1992	Selangor	DM	83	16	19.3	Diarrhea								Lai, 1992 [41]
3	Children with cancer	1996–1997	Kelantan	DM	50	2	4	Diarrhea								Menon et al., 1999 [59]
4	Dengue patients	2015–2016	Selangor	PID	89	21	23.6	Gastrointestinal symptoms								Thergarajan et al., 2019 [60]
5	Cancer patients	2008	Selangor	PID	311	24	7.7	Not reported	3	1	11	5	0	0	0	Tan et al., 2009 [61]
6	HIV patients	2008	Selangor	PID	247	49	19.8	Not reported	2	1	9	6	0	0	0	Tan et al., 2009 [61]
7	Colorectal cancer patients	2010–2012	Selangor	PID	204	43	21.08	Gastrointestinal symptoms	6	2	7	0	1	0	6	Kumarasamy et al., 2014 [15]
8	Acute diarrhea and hospitalized	2004	Perak	DM	27	1	3.7	Gastrointestinal symptoms								Hakim et al., 2007 [49]
9	Diarrhea but not hospitalized	2004	Perak	DM	24	2	8.3	Gastrointestinal symptoms								Hakim et al., 2007 [49]
	**High Risk Group**															
1	Animal handlers	1998	Selangor	DM	105	43	41	Not reported								Salim et al., 1999 [57]
2	Migrant workers	2014–2015	Selangor	PID	212	68	30.9	Gastrointestinal symptoms	8	2	12	0	0	0	0	Sahimin et al., 2020 [62]
3	Prisoners	2012–2013	Selangor	PID	294	43	14.6	Gastrointestinal symptoms	7	0	25	0	0	1	0	Angal et al., 2015 [63]

PID: Detection of *Blastocystis* via PCR, in vitro cultivation, and direct stool smear. DM: Detection of *Blastocystis* via direct microscopy.

## Data Availability

The full data supporting this systematic review are available in the included studies in the References section. The analyzed data presented in this study are available in Table S1.

## Supplementary Materials

The following supporting information can be downloaded at: https://www.mdpi.com/article/10.3390/tropicalmed8080415/s1, Table S1: Data extraction table.

## References

[1] Lee H., Yoon Y. (2021). Etiological agents implicated in foodborne illness worldwide. Food Sci. Anim. Resour..

[2] Chen J., Ding W., Li Z., Zhou D.D., Yang P., Wang R.B., Zheng B., Sheng H.F., Guan Y.Y., Xiao N. (2020). From parasitic disease control to global health: New orientation of the National Institute of Parasitic Diseases, China CDC. Acta Trop..

[3] Chelkeba L., Mekonnen Z., Alemu Y., Emana D. (2020). Epidemiology of intestinal parasitic infections in preschool and school-aged Ethiopian children: A systematic review and meta-analysis. BMC Public Health.

[4] Osafo R., Balali G.I., Amissah-Reynolds P.K., Gyapong F., Addy R., Nyarko A.A., Wiafe P. (2022). Microbial and parasitic contamination of vegetables in developing countries and their food safety guidelines. J. Food Qual..

[5] Stenzel D., Boreham P. (1996). *Blastocystis hominis* revisited. Clin. Microbiol. Rev..

[6] Tan K.S., Singh M., Yap E.H. (2002). Recent advances in *Blastocystis hominis* research: Hot spots in terra incognita. Int. J. Parasitol..

[7] Skotarczak B. (2018). Genetic diversity and pathogenicity of *Blastocystis*. Ann. Agric. Environ. Med..

[8] Paniker C.J., Ghosh S. (2017). Paniker’s Textbook of Medical Parasitology.

[9] Ajjampur S.S., Tan K.S. (2016). Pathogenic mechanisms in *Blastocystis* spp.—Interpreting results from in vitro and in vivo studies. Parasitol. Int..

[10] Clark C.G. (1997). Extensive genetic diversity in *Blastocystis hominis*. Mol. Biochem. Parasitol..

[11] Zanetti A.D.S., Malheiros A.F., De Matos T.A., Longhi F.G., Moreira L.M., Silva S.L., Castrillon S.K.I., Ferreira S.M.B., Ignotti E., Espinosa O.A. (2020). Prevalence of *Blastocystis* sp. infection in several hosts in Brazil: A systematic review and meta-analysis. Parasit. Vectors.

[12] Kataki M.M., Tavalla M., Beiromvand M. (2019). Higher prevalence of *Blastocystis hominis* in healthy individuals than patients with gastrointestinal symptoms from Ahvaz, southwestern Iran. Comp. Immunol. Microbiol. Infect. Dis..

[13] Yakoob J., Abbas Z., Khan R., Tariq K., Awan S., Beg M.A. (2018). Association of *Helicobacter pylori* and protozoal parasites in patients with chronic diarrhoea. Br. J. Biomed. Sci..

[14] Kesuma Y., Firmansyah A., Bardosono S., Sari I.P., Kurniawan A. (2019). *Blastocystis* ST-1 is associated with irritable bowel syndrome-diarrhoea (IBS-D) in Indonesian adolescences. Parasite Epidemiol. Control.

[15] Kumarasamy V., Roslani A.C., Rani K.U., Govind S.K. (2014). Advantage of using colonic washouts for *Blastocystis* detection in colorectal cancer patients. Parasit. Vectors.

[16] Jiménez P.A., Jaimes J.E., Ramírez J.D. (2019). A summary of *Blastocystis* subtypes in North and South America. Parasit. Vectors.

[17] Khorshidvand Z., Khazaei S., Amiri M., Taherkhani H., Mirzaei A. (2021). Worldwide prevalence of emerging parasite *Blastocystis* in immunocompromised patients: A systematic review and meta-analysis. Microb. Pathog..

[18] Kumarasamy V., Anbazhagan D., Subramaniyan V., Vellasamy S. (2018). *Blastocystis* sp., parasite associated with gastrointestinal disorders: An overview of its pathogenesis, immune modulation and therapeutic strategies. Curr. Pharm. Des..

[19] Légeret C., Rüttimann C., Furlano R.I., Ruf T., Poppert S., Fankhauser H., Köhler H. (2020). *Blastocystis* in Swiss children: A practical approach. Eur. J. Pediatr..

[20] Robles-Cabrera M.X., Maguiña J.L., Gonzales-Huerta L., Panduro-Correa V., Dámaso-Mata B., Pecho-Silva S., Navarro-Solsol A.C., Rabaan A.A., Rodríguez-Morales A.J., Arteaga-Livias K. (2021). *Blastocystis* species and gastrointestinal symptoms in Peruvian adults attended in a public hospital. Infect. Chemother..

[21] Yowang A., Tsaousis A.D., Chumphonsuk T., Thongsin N., Kullawong N., Popluechai S., Gentekaki E. (2018). High diversity of *Blastocystis* subtypes isolated from asymptomatic adults living in Chiang Rai, Thailand. Infect. Genet. Evol..

[22] Qi M., Wei Z., Zhang Y., Zhang Q., Li J., Zhang L., Wang R. (2020). Genetic diversity of *Blastocystis* in kindergarten children in southern Xinjiang, China. Parasit. Vectors.

[23] Maloney J.G., da Cunha M.J., Molokin A., Cury M.C., Santin M. (2021). Next-generation sequencing reveals wide genetic diversity of *Blastocystis* subtypes in chickens including potentially zoonotic subtypes. Parasitol. Res..

[24] Popruk S., Adao D.E.V., Rivera W.L. (2021). Epidemiology and subtype distribution of *Blastocystis* in humans: A review. Infect. Genet. Evol..

[25] Ramírez J.D., Sánchez L.V., Bautista D.C., Corredor A.F., Flórez A.C., Stensvold C.R. (2014). *Blastocystis* subtypes detected in humans and animals from Colombia. Infect. Genet. Evol..

[26] Deng L., Chai Y., Zhou Z., Liu H., Zhong Z., Hu Y., Fu H., Yue C., Peng G. (2019). Epidemiology of *Blastocystis* sp. infection in China: A systematic review. Parasite.

[27] Salehi R., Haghighi A., Stensvold C.R., Kheirandish F., Azargashb E., Raeghi S., Kohansal C., Bahrami F. (2017). Prevalence and subtype identification of *Blastocystis* isolated from humans in Ahvaz, Southwestern Iran. Gastroenterol Hepatol. Bed. Bench.

[28] Scanlan P.D., Stensvold C.R., Cotter P.D. (2015). Development and application of a *Blastocystis* subtype-specific PCR assay reveals that mixed-subtype infections are common in a healthy human population. Appl. Environ. Microbiol..

[29] Osorio-Pulgarin M.I., Higuera A., Beltran-Álzate J.C., Sánchez-Jiménez M., Ramírez J.D. (2021). Epidemiological and molecular characterization of *Blastocystis* infection in children attending daycare centers in Medellín, Colombia. Biology.

[30] Souppart L., Moussa H., Cian A., Sanciu G., Poirier P., El Alaoui H., Delbac F., Boorom K., Delhaes L., Dei-Cas E. (2010). Subtype analysis of *Blastocystis* isolates from symptomatic patients in Egypt. Parasitol. Res..

[31] Moosavi A., Haghighi A., Mojarad E.N., Zayeri F., Alebouyeh M., Khazan H., Kazemi B., Zali M.R. (2012). Genetic variability of *Blastocystis* sp. isolated from symptomatic and asymptomatic individuals in Iran. Parasitol. Res..

[32] Ahmed S.A., El-Mahallawy H.S., Mohamed S.F., Angelici M.C., Hasapis K., Saber T., Karanis P. (2022). Subtypes and phylogenetic analysis of *Blastocystis* sp. isolates from West Ismailia, Egypt. Sci. Rep..

[33] Aykur M., Calıskan Kurt C., Dirim Erdogan D., Biray Avcı C., Vardar R., Aydemir S., Girginkardesler N., Gunduz C., Dagci H. (2023). Distribution and Phylogenetic Analysis of Subtypes and Alleles of *Blastocystis* sp. in the Stool Samples Collected from Patients with Gastrointestinal Complaints in İzmir, Turkey. Acta Parasitol..

[34] Abu A., Sutthikornchai C., Mahittikorn A., Koompapong K., Chiabchalard R., Arthan D., Soonthornworasiri N., Popruk S. (2023). Prevalence and Subtype Distribution of *Blastocystis* Isolated from School-Aged Children in the Thai-Myanmar Border, Ratchaburi Province, Thailand. Int. J. Environ. Res. Public Health.

[35] Nithyamathi K., Chandramathi S., Kumar S. (2016). Predominance of *Blastocystis* sp. infection among school children in Peninsular Malaysia. PLoS ONE.

[36] Chen H., Hao Y., Liu Y., Xu M., Zhang W., Li H., Yang F. (2023). The frequency and subtype distribution of *Blastocystis* sp. in humans and domestic animals in households in Heilongjiang Province, China. Acta Trop..

[37] Rudzińska M., Kowalewska B., Kurpas M., Szostakowska B. (2022). Rare occurrence of *Blastocystis* in pet animals and their owners in the Pomeranian Voivodeship in Poland in the light of literature data. J. Clin. Med..

[38] Anuar T.S., Ghani M.K.A., Azreen S.N., Salleh F.M., Moktar N. (2013). *Blastocystis* infection in Malaysia: Evidence of waterborne and human-to-human transmissions among the Proto-Malay, Negrito and Senoi tribes of Orang Asli. Parasit. Vectors.

[39] Asma I., Johari S., Sim B.L., Lim Y.A. (2011). How common is intestinal parasitism in HIV-infected patients in Malaysia?. Trop. Biomed..

[40] Moher D., Liberati A., Tetzlaff J., Altman D.G., Group P. (2010). Preferred reporting items for systematic reviews and meta-analyses: The PRISMA statement. PLoS Med..

[41] Lai K.P. (1992). Intestinal protozoan infections in Malaysia. Southeast Asian J. Trop. Med. Public Health.

[42] Noradilah S.A., Moktar N., Anuar T.S., Lee I.L., Salleh F.M., Manap S.N.A.A., Nordin A., Mohtar N.S.H.M., Azrul S.M., Abdullah W.O. (2017). Molecular epidemiology of Blastocystosis in Malaysia: Does seasonal variation play an important role in determining the distribution and risk factors of *Blastocystis* subtype infections in the Aboriginal community?. Parasit. Vectors.

[43] Mohammad N.A., Al-Mekhlafi H.M., Moktar N., Anuar T.S. (2017). Prevalence and risk factors of *Blastocystis* infection among underprivileged communities in rural Malaysia. Asian Pac. J. Trop. Med..

[44] Mohammad N.A., Al-Mekhlafi H.M., Anuar T.S. (2018). Subtype distribution of *Blastocystis* isolated from humans and associated animals in an indigenous community with poor hygiene in Peninsular Malaysia. Trop. Biomed..

[45] Mohammad N.A., Al-Mekhlafi H.M., Anuar T.S. (2018). Genetic diversity of *Blastocystis* isolates from symptomatic and asymptomatic Orang Asli In Pahang, Malaysia. Southeast Asian J. Trop. Med..

[46] Noor Azian M.Y., San Y.M., Gan C.C., Yusri M.Y., Nurulsyamzawaty Y., Zuhaizam A.H., Maslawaty M.N., Norparina I., Vythilingam I. (2007). Prevalence of intestinal protozoa in an aborigine community in Pahang, Malaysia. Trop. Biomed..

[47] Sinniah B., Sabaridah I., Soe M., Sabitha P., Awang I., Ong G., Hassan A. (2012). Determining the prevalence of intestinal parasites in three Orang Asli (Aborigines) communities in Perak, Malaysia. Trop. Biomed..

[48] Gee H.T.S., Kamel M.A.G. (2020). Intestinal protozoan infections of schoolchildren in an Aboriginal (Orang Asli) settlement in Perak, Malaysia. Int. Med. J..

[49] Hakim S.L., Gan C.C., Malkit K., Azian M.N., Chong C.K., Shaari N., Zainuddin W., Chin C.N., Sara Y., Lye M.S. (2007). Parasitic infections among orang asli (aborigine) in the Cameron Highlands, Malaysia. Southeast Asian J. Trop. Med..

[50] Elyana F.N., Al-Mekhlafi H.M., Ithoi I., Abdulsalam A.M., Dawaki S., Nasr N.A., Atroosh W.M., Abd-Basher M.H., Al-Areeqi M.A., Sady H. (2016). A tale of two communities: Intestinal polyparasitism among Orang Asli and Malay communities in rural Terengganu, Malaysia. Parasit. Vectors.

[51] Abdulsalam A.M., Ithoi I., Al-Mekhlafi H.M., Ahmed A., Surin J., Mak J.W. (2012). Drinking water is a significant predictor of *Blastocystis* infection among rural Malaysian primary schoolchildren. Parasitology.

[52] Sinniah B., Rajeswari B. (1994). *Blastocystis hominis* infection, a cause of human diarrhea. Southeast Asian J. Trop. Med. Public Health.

[53] Abd Ghani M.K., Alharazi T. (2016). Blastocystosis amongst the Orang Asli (Aborigine) schoolchildren at Pos Senderut, Pahang, Malaysia. Int. Med. J..

[54] Kamel M.A.G., Najah F. (2021). Blastocystosis amongst the Orang Asli (Aborigine) schoolchildren at Pos Senderut, Kuala Lipis, Malaysia. Int. Med. J..

[55] Al-Delaimy A.K., Al-Mekhlafi H.M., Nasr N.A., Sady H., Atroosh W.M., Nashiry M., Anuar T.S., Moktar N., Lim Y.A.L., Mahmud R. (2014). Epidemiology of intestinal polyparasitism among Orang Asli school children in rural Malaysia. PLoS Neglected Trop. Dis..

[56] Adli M.N., Kamel M.A.G. (2020). Blastocystosis amongst the Orang Asli (Aborigine) school children of SKTAR Kuala Kubu Bharu, Selangor, Malaysia. Int. Med. J..

[57] Salim H.R., Kumar G.S., Vellayan S., Mak J., Anuar A.K., Init I., Vennila G., Saminathan R., Ramakrishnan K. (1999). *Blastocystis* in animal handlers. Parasitol. Res..

[58] Suresh K., Salim H.R., Jamaiah I., Anuar A.K. (2001). *Blastocystis hominis* in high-rise flat dwellers in Kuala Lumpur, Malaysia. Trans. R. Soc. Trop..

[59] Menon B.S., Abdullah M.S., Mahamud F., Singh B. (1999). Intestinal parasites in Malaysian children with cancer. J. Trop. Pediatr..

[60] Thergarajan G., Kumar S., Bhassu S., Omar S.F.B.S., Rampal S. (2019). Effect of *Blastocystis* sp. in dengue patients—Increase in the treatment cost and exacerbation of symptoms. PLoS ONE.

[61] Tan T., Ong S., Suresh K. (2009). Genetic variability of *Blastocystis* sp. isolates obtained from cancer and HIV/AIDS patients. Parasitol. Res..

[62] Sahimin N., Meor Termizi F.H., Rajamanikam A., Mohd Nazri N.A., Govind S.K., Mohd Zain S.N. (2020). Prevalence and subtypes of *Blastocystis* among migrant workers from different working sectors in Peninsular Malaysia. Parasitol. Res..

[63] Angal L., Mahmud R., Samin S., Yap N.J., Ngui R., Amir A., Ithoi I., Kamarulzaman A., Lim Y.A.L. (2015). Determining intestinal parasitic infections (IPIs) in inmates from Kajang Prison, Selangor, Malaysia for improved prison management. BMC Infect. Dis..

[64] Arisue N., Hashimoto T., Yoshikawa H., Nakamura Y., Nakamura G., Nakamura F., Yano T.A., Hasegawa M. (2002). Phylogenetic position of *Blastocystis hominis* and of stramenopiles inferred from multiple molecular sequence data. J. Eukaryot. Microbiol..

[65] Scanlan P.D., Stensvold C.R. (2013). *Blastocystis*: Getting to grips with our guileful guest. Trends Parasitol..

[66] El Safadi D., Gaayeb L., Meloni D., Cian A., Poirier P., Wawrzyniak I., Delbac F., Dabboussi F., Delhaes L., Seck M. (2014). Children of Senegal River Basin show the highest prevalence of *Blastocystis* sp. ever observed worldwide. BMC Infect. Dis..

[67] Pandey P.K., Verma P., Marathe N., Shetty S., Bavdekar A., Patole M.S., Stensvold C.R., Shouche Y.S. (2015). Prevalence and subtype analysis of *Blastocystis* in healthy Indian individuals. Infect. Genet. Evol..

[68] Gabrielli S., Furzi F., Sulekova L.F., Taliani G., Mattiucci S. (2020). Occurrence of *Blastocystis*-subtypes in patients from Italy revealed association of ST3 with a healthy gut microbiota. Parasite Epidemiol. Control.

[69] Nemati S., Reza Zali M., Johnson P., Mirjalali H., Karanis P. (2021). Molecular prevalence and subtype distribution of *Blastocystis* sp. in Asia and Australia. J. Water Health.

[70] Rauff-Adedotun A.A., Meor Termizi F.H., Shaari N., Lee I.L. (2021). The Coexistence of *Blastocystis* spp. in Humans, Animals and Environmental Sources from 2010–2021 in Asia. Biology.

[71] Noël C., Dufernez F., Gerbod D., Edgcomb V.P., Delgado-Viscogliosi P., Ho L.C., Singh M., Wintjens R., Sogin M.L., Capron M. (2005). Molecular phylogenies of *Blastocystis* isolates from different hosts: Implications for genetic diversity, identification of species, and zoonosis. J. Clin. Microbiol..

[72] Asghari A., Sadeghipour Z., Hassanipour S., Abbasali Z., Ebrahimzadeh-Parikhani H., Hashemzaei M., Alimardani V., Hatam G. (2021). Association between *Blastocystis* sp. infection and immunocompromised patients: A systematic review and meta-analysis. Environ. Sci. Pollut. Res..

[73] Kumarasamy V., Kuppusamy U.R., Samudi C., Kumar S. (2013). *Blastocystis* sp. subtype 3 triggers higher proliferation of human colorectal cancer cells, HCT116. Parasitol. Res..

[74] Kumarasamy V., Kuppusamy U.R., Jayalakshmi P., Samudi C., Ragavan N.D., Kumar S. (2017). Exacerbation of colon carcinogenesis by *Blastocystis* sp.. PLoS ONE.

[75] Deng L., Wojciech L., Gascoigne N.R.J., Peng G., Tan K.S.W. (2021). New insights into the interactions between *Blastocystis*, the gut microbiota, and host immunity. PLoS Pathogens.

[76] Basak S., Rajurkar M.N., Mallick S.K. (2014). Detection of *Blastocystis hominis: A* controversial human pathogen. Parasitol. Res..

[77] Vennila G., Suresh Kumar G., Khairul Anuar A., Rajah S., Saminathan R., Sivanandan S., Ramakrishnan K. (1999). Irregular shedding of *Blastocystis hominis*. Parasitol. Res..

[78] Öner T.Ö., Karabey M., Can H., Döşkaya A.D., Karakavuk M., Gül A., Gökmen A.A. (2022). Molecular investigation of *Blastocystis* sp. and its subtypes in cancer patients under chemotherapy in Aegean region, Turkey. Acta. Trop..

[79] Wakid M.H., Aldahhasi W.T., Alsulami M.N., El-Kady A.M., Elshabrawy H.A. (2022). Identification and genetic characterization of *Blastocystis* species in patients from Makkah, Saudi Arabia. Infect. Drug Resist..

[80] Kim M.J., Lee Y.J., Kim T.J., Won E.J. (2021). Gut microbiome profiles in colonizations with the enteric protozoa *Blastocystis* in Korean populations. Microorganisms.

[81] Kim M.J., Won E.J., Kim S.H., Shin J.H., Chai J.Y. (2020). Molecular detection and subtyping of human *Blastocystis* and the clinical implications: Comparisons between diarrheal and non-diarrheal groups in Korean populations. Korean J. Parasitol..

[82] Javanmard E., Niyyati M., Ghasemi E., Mirjalali H., Aghdaei H.A., Zali M.R. (2018). Impacts of human development index and climate conditions on prevalence of *Blastocystis*: A systematic review and meta-analysis. Acta Trop..

[83] Leelayoova S., Siripattanapipong S., Thathaisong U., Naaglor T., Taamasri P., Piyaraj P., Mungthin M. (2008). Drinking water: A possible source of *Blastocystis* spp. subtype 1 infection in schoolchildren of a rural community in central Thailand. Am. J. Trop. Med. Hyg..

[84] Lee I.L., Tan T.C., Tan P.C., Nanthiney D.R., Biraj M.K., Surendra K.M., Suresh K.G. (2012). Predominance of *Blastocystis* sp. subtype 4 in rural communities, Nepal. Parasitol. Res..

[85] Rashid M.F.A. (2017). Characteristics, trends and spatial distribution of urban migration in Malaysia: A case study of the Klang Valley region. UPLanD.

[86] Sharif M., Daryani A., Kia E., Rezaei F., Nasiri M., Nasrolahei M. (2015). Prevalence of intestinal parasites among food handlers of Sari, Northern Iran. Rev. Inst. Med. Trop..

[87] Abu-Madi M., Aly M., Behnke J.M., Clark C.G., Balkhy H. (2015). The distribution of *Blastocystis* subtypes in isolates from Qatar. Parasit. Vectors.

[88] Greige S., El Safadi D., Bécu N., Gantois N., Pereira B., Chabé M., Benamrouz-Vanneste S., Certad G., El Hage R., Chemaly M. (2018). Prevalence and subtype distribution of *Blastocystis* sp. isolates from poultry in Lebanon and evidence of zoonotic potential. Parasit. Vectors.

[89] Pintong A.R., Sunyanusin S., Prasertbun R., Mahittikorn A., Mori H., Changbunjong T., Komalamisra C., Sukthana Y., Popruk S. (2018). *Blastocystis* subtype 5: Predominant subtype on pig farms, Thailand. Parasitol. Int..

[90] Parkar U., Traub R.J., Vitali S., Elliot A., Levecke B., Robertson I., Geurden T., Steele J., Drake B., Thompson R.C. (2010). Molecular characterization of *Blastocystis* isolates from zoo animals and their animal-keepers. Vet. Parasitol..

[91] Sanggari A., Komala T., Rauff-Adedotun A.A., Awosolu O.B., Attah O.A., Farah Haziqah M.T. (2022). Blastocystis in captivated and free-ranging wild animals worldwide: A review. Trop. Biomed..

[92] Mokhtar A., Youssef A. (2018). Subtype analysis of *Blastocystis* spp. isolated from domestic mammals and poultry and its relation to transmission to their in-contact humans in Ismailia governorate, Egypt. Parasitol. United J..

[93] Chandramathi S., Suresh K., Anita Z.B., Kuppusamy U.R. (2012). Infections of *Blastocystis hominis* and microsporidia in cancer patients: Are they opportunistic?. Trans. R. Soc. Trop. Med. Hyg..

[94] Bednarska M., Jankowska I., Pawelas A., Piwczyńska K., Bajer A., Wolska-Kuśnierz B., Wielopolska M., Welc-Falęciak R. (2018). Prevalence of *Cryptosporidium*, *Blastocystis*, and other opportunistic infections in patients with primary and acquired immunodeficiency. Parasitol. Res..

[95] Puthia M.K., Lu J., Tan K.S. (2008). *Blastocystis ratti* contains cysteine proteases that mediate interleukin-8 response from human intestinal epithelial cells in an NF-κB-dependent manner. Eukaryot. Cell.

[96] Puthia M.K., Vaithilingam A., Lu J., Tan K.S. (2005). Degradation of human secretory immunoglobulin A by *Blastocystis*. Parasitol. Res..

[97] Alfellani M.A., Stensvold C.R., Vidal-Lapiedra A., Onuoha E.S.U., Fagbenro-Beyioku A.F., Clark C.G. (2013). Variable geographic distribution of *Blastocystis* subtypes and its potential implications. Acta Trop..

[98] Ben Abda I., Maatoug N., Ben Romdhane R., Bouhelmi N., Zallegua N., Aoun K., Viscogliosi E., Bouratbine A. (2017). Prevalence and Subtype Identification of *Blastocystis* sp. in Healthy Individuals in the Tunis Area, Tunisia. Am. J. Trop. Med. Hyg..

[99] Wong K.H., Ng G.C., Lin R.T., Yoshikawa H., Taylor M.B., Tan K.S. (2008). Predominance of subtype 3 among *Blastocystis* isolates from a major hospital in Singapore. Parasitol. Res..

[100] Malheiros A.F., Stensvold C.R., Clark C.G., Braga G.B., Shaw J.J. (2011). Short report: Molecular characterization of *Blastocystis* obtained from members of the indigenous Tapirapé ethnic group from the Brazilian Amazon region, Brazil. Am. J. Trop. Med. Hyg..

[101] Cian A., El Safadi D., Osman M., Moriniere R., Gantois N., Benamrouz-Vanneste S., Delgado-Viscogliosi P., Guyot K., Li L.L., Monchy S. (2017). Molecular epidemiology of *Blastocystis* sp. in various animal groups from two French zoos and evaluation of potential zoonotic risk. PLoS ONE.

[102] Lim Y.A., Ngui R., Shukri J., Rohela M., Mat Naim H.R. (2008). Intestinal parasites in various animals at a zoo in Malaysia. Vet. Parasitol..

[103] Wawrzyniak I., Poirier P., Viscogliosi E., Dionigia M., Texier C., Delbac F., Alaoui H.E. (2013). *Blastocystis*, an unrecognized parasite: An overview of pathogenesis and diagnosis. Ther. Adv. Infect. Dis..

[104] Roberts T., Stark D., Harkness J., Ellis J. (2014). Update on the pathogenic potential and treatment options for *Blastocystis* sp.. Gut Pathog..

[105] Rajamanikam A., Hooi H.S., Kudva M., Samudi C., Govind S.K. (2022). Distinct phenotypic variation of *Blastocystis* sp. ST3 from urban and Orang Asli population—An influential consideration during sample collection in surveys. Biology.

