# Supplementary material for: Systematic Review and Meta-Analysis: Epidemiology of Human Blastocystis spp. Infection in Malaysia
Source: Trop Med Infect Dis. 2023 Aug 15;8(8):415. doi: 10.3390/tropicalmed8080415 (PMC10457748; doi:10.3390/tropicalmed8080415)
Supplement: Supplementary file 1 [file tropicalmed-08-00415-s001.zip › tropicalmed-2518742-supplementary.pdf]

Table S1: Data extraction table

No	Author & Year	Sampling	Setting	Method	Main results	Conclusion	Scoring
1	Kumarasamy et al., 2014 [15]	Patients who underwent diagnostic colonoscopy in University of Malaya Medical Centre (UMMC)	Department of Parasitology, University of Malaya	Objective: to detect the prevalence *Blastocystis* in colonic washouts and stool samples of CRC patients.<br><br>Colonic washouts were obtained at the time of (or immediately prior to) the diagnosis of CRC. Whereas, faecal samples were obtained via the standard clinical procedures whereby patients will be given a duration of 1–2 weeks to deliver their faecal samples. Colonic washouts were collected in clean disposable bowls. Each sample was centrifuged at 1,400 × g and the pellets obtained were cultured in Jones' medium supplemented with 10% horse serum and incubated at 37°C for 24 h and then screened for Blastocystis. Fresh colonic washout and faecal samples were routinely processed by the formal ether concentration technique (FECT) to obtain stool concentrate PCR technique was used to detect *Blastocystis* in addition to standard stool culture technique in both colonic washouts and faecal samples.	A total of 43 (21.08%) samples were positive for *Blastocystis* infection in CRC patients and was significantly higher compared to normal individuals (n = 22, 9.95%, p <0.01 Overall, ST3 was the most prevalent subtype (n = 30, 14.71%), whereas ST1, ST2, and ST5 were seen in 5.39% (n = 11), 3.43% (n = 7) and 0.49% (n = 1) of the CRC patients, respectively. Besides that, mixed subtype infections were detected in six samples which were 0.98% (n = 2, ST1 and ST2) and 1.96% (n = 4, ST2 and ST3). ST3 infection was also found to be statistically significant in CRC patients as compared with the control group.	Colonic washouts can be a better alternative to faecal samples to examine for Blastocystis infection especially in CRC cases. Our study shows that Blastocystis infection is common in CRC patients and it indicates subtype 3 as predominant among these individuals.	6
2	Nithyamathi et al., 2016 [35]	1760, 26 schools throughout the country	Department of Parasitology, Faculty of Medicine, University of Malaya, Kuala Lumpur, Malaysia	Objective: To investigate the prevalence of intestinal parasitic infections among urban and rural school children from five states namely Selangor, Perak, Pahang, Kedah and Johor in Peninsula Malaysia.<br><br>Faecal examination was performed by adding approximately pea size of faecal sample into Jones' medium supplemented with 10% horse serum, incubated at 37°C and examined using light microscope for the subsequent 48 to 72 hours. Direct faecal examination and formal ether concentration techniques were used to examine the presence of other intestinal parasites using light microscope, *in vitro* cultivation in Jones's medium and identified by light microscopy were subjected to DNA extraction using a commercial kit.	The total prevalence was 10.6%). Perak had the highest infection. Subtype (ST) 3 (54.3%) is the most predominant ST with persons infected with only ST1 and ST3 showing symptoms. *Blastocystis* infection significantly associated with low household income, low parent's education and presence of symptoms.	It is critical that we institute deworming and treatment to eradicate the parasite especially in rural school children	6
3	Anuar et al., 2013 [39]	Three Orang Asli tribes (Proto-Malay, Negrito and Senoi) in selected villages at Negeri Sembilan, Perak and Pahang, Peninsular Malaysia.	Department of Parasitlogy, Faculty of Medicine, Universiti Kebangsaan Malaysia	Objective: to contribute to a better comprehension of the epidemiology of this infection, a cross-sectional survey aimed at providing the first documented data on the prevalence and risk factors associated with *Blastocystis* infection.<br><br>A structured questionnaire was used. The questionnaire was pre-tested among Orang Asli individuals who were admitted to Gombak Hospital, Selangor state. Trained research assistants interviewed participants in person, asking questions for demographic data (i.e., age, gender and education level), socioeconomic background (i.e., occupation, household income and educational status)	Of 500 individuals, 20.4% (102) were detected positive for *Blastocystis*; 13.3% (20/150) of Proto-Malays, 21.6% (30/139) of Negritos and 24.7% (52/211) of Senois were positive for *Blastocystis*, respectively. The positive cases showed a decrease with increasing age and most of the positive cases were observed in individuals less than 15 years old. Multivariate analysis confirmed that drinking untreated water and the presence of other family members infected with *Blastocystis* were significant risk factors of infection among the three tribes and overall population studied.	*Blastocystis* infection is prevalent among Orang Asli communities in Malaysia. The infection may be transmitted through waterborne and humanto-human contact. Therefore, interventions with the provision of clean water supply for the communities and health education especially to the parents are urgently required	6
4	Lai, 1992 [41]	A total of 7995 samples with 50% response rate from 8 parts of Peninsular Malaysia and two in Sarawak. Routine examination of chronic diarrheic cases (1990-1992)-83 cases. Study included different age groups, sex and ethnicity	Institute for Medical Research, Kuala Lumpur	Objective: To identify prevalence of protozoan parasites infections in Malaysia.<br><br>Stools were collected daily and the samples were fixed in polyvinyl alcohol immediately in the field. Smears of stool samples were then stained in trichrome and examined for intestinal protozoa in the laboratory.	The total prevalence was 5-9.2% among the survey cases and 19.3% among chronic diarrheic case	The other protozoon of interest is *Blastocystis* which had a high incidence, mainly among chronic diarrheic cases received in our laboratory over a two-year period. It is still being debated whether this protozoon is a causative agent of diarrhoea.	3
5	Noradilah et al., 2017 [42]	A total of 473 faecal samples were collected from aborigines	Department of Parasitology and Medical Entomology, Faculty of Medicine, Universiti Kebangsaan Malaysia Medical Centre.	Objective: To provide the prevalence of *Blastocystis* and to determine the potential risk factors associated with each subtype during the wet and dry seasons in the Aboriginal community, Pahang, Malaysia.<br><br>Structured questionnaires used. All faecal samples were subjected to genomic DNA extraction using QIAamp® Fast DNA Stool Mini Kit (QIAGEN, Hilden, Germany), as per the manufacturer's instructions. Screening of the samples was performed using the primers.	The total prevalence was 42.6% and 37.8% positive for Blastocystis ST1, ST2, ST3 and ST4 during wet and dry seasons, respectively. Prevalence of Blastocystis ST1 was significantly higher during the wet season compared to the dry season (Z = 2.146, P < 0.05)<br><br>Analysis of the association of each Blastocystis subtype with socioeconomic characteristics showed the presence of other family members infected with Blastocystis ST3 and the use of stored river water for domestic activities were the significant risk factors for Blastocystis ST3 infections during both seasons.<br><br>Untreated water supply and low monthly household income (less or equal to RM 500) were the other significant risk factors for Blastocystis ST3 infections during wet and dry season, respectively. The presence of other family members with Blastocystis ST1 and ST2 was the only significant risk factor associated with ST1 and ST2 infections during both seasons.	Transmission of *Blastocystis* ST1, ST2 and ST3 occurred from person to person during both seasons. The waterborne transmission was also identified as a mode of transmission of *Blastocystis* ST3.	6
6	Mohammad et al., 2017 [43]	Cross-sectional study<br>Sample size: 253<br>Age: 1 and 85 years Sungai Lembing 2 villages (Sungai Mas and Sungai Jin)	Centre of Medical Laboratory Technology, Faculty of Health Sciences, Universiti Teknologi MARA, Puncak Alam Campus, Selangor, Malaysia	Objective: To determine the prevalence and risk factors of *Blastocystis* among underprivileged communities living in rural Malaysia.<br><br>Stool samples were examined using Wheatley's trichrome stain after *in-vitro* cultivation in Jones' medium to detect the presence of *Blastocystis*. Dissemination of containers with 100 mL wide mouth screw-capped and pre-labelled with participant's name and identification number to each participant for stool sample collection were done after administrating the questionnaire. The coverslip was mounted using Distrene, Plasticiser and Xylene (DPX) and observed under a light microscope at magnifications of ×100. *In-vitro* cultivation of *Blastocystis*	The total prevalence was 40.7%. Associated factors: age 15 years and presence of infected family members.	Blastocystosis is revealed through this study to be still prevalent among Orang Asli communities in rural Malaysia. Screening of other family members and giving treatment to the infected individuals are crucial to end the transmission. health education on good personal and food hygiene practices are provided in order to reduce the morbidity and transmission of *Blastocystis* infection.	4
7	Mohammad et al., 2018 [44]	Sample size: 243<br>Age: 2-70 Years<br>two villages namely Sungai Mas and Sungai Jin	Centre of Medical Laboratory Technology, Faculty of Health Sciences, Universiti Teknologi MARA, Puncak Alam Campus, Selangor, Malaysia	Objective: To identify prevalence of *Blastocystis* among rural population<br><br>DNA extraction from stool samples; DNA amplification by PCR; DNA sequencing and phylogenetic; Reconstruction; Chi-square	The prevalence: 18.5%. Genders and age groups are significantly associated with the prevalence. Subtypes: ST3 was the predominant subtype, followed by ST1 and ST2.	There is various host-specific subtypes in the lifecycle of *Blastocystis*	3
8	Mohammad et al., 2018 [45]	Study design: cross-sectional study<br>Sample size: 253<br>Age: 2 to 56 years.<br>Sungai Lembing (3°55'N, 103°02'E), Pahang state, Malaysia	Centre of Medical Laboratory Technology, Faculty of Health Sciences, Universiti Teknologi MARA, Puncak Alam Campus, Selangor, Malaysia	Objective: To identify prevalence of *Blastocystis* among rural population<br><br>Data collection: via questionnaire (demographic data, socioeconomic, signs and symptoms and medical treatment). Microscopic examination and molecular were performed. *In vitro* cultivation of *Blastocystis*	The prevalence of *Blastocystis* was 26.4% (microscopy-positive for *Blastocystis*) and 17.7%. PCR-positive samples.	Infection was likely not associated with specific subtypes, even if some subtypes were predominant in the epidemiologic studies, but rather with a conjunction of host factors, such as immune status and age.	3
9	Noor Azian et al., 2007 [46]	130 stool samples collected from Pos Senderut, 7 villages Kg Regang, Kg	Institute for Medical Research, Kuala Lumpur	Objective: To identify prevalence of *Blastocystis* among rural population	The total prevalence was (52.3%). Although, the infection was more frequent in females than males it was not statistically significant (P=0.144). The	The high prevalence was attributable to poor environmental management, poor personal hygiene and lack of health education	4

		Muman, Kg Sebeng, Kg Kuala Senderut,<br>Pos Senderut, Kg Poyan and Kg Bukit Long.		Detection of intestinal protozoa was based on morphological characteristic of specific protozoa. The specimens were immediately fixed upon arrival at the temporary laboratory set-up in the field. The fixatives were Polyvinyl Alcohol (PVA) solution. The preserved samples were brought to Parasitology Unit, Institute for Medical Research (IMR) for processing and analysis. The Trichrome staining technique was employed for the PVA preserved stools. These PVA preservatives were mainly to detect cyst and trophozoite forms of intestinal protozoa.	highest infection rate of infection was in the 1-10 years old age group (45.4%) and the lowest infection rate of infection was the 61-70 years age group (3.8%). it was not statistically significant.		
10	Sinniah et al., 2012 [47]	The target population were children and adult Orang Asli (Aborigines) from different locations in Perak.	Universiti Kuala Lumpur Royal College of Medicine Perak, Ipoh, Perak, Malaysia	Objective: To determine the prevalence of intestinal parasites among children and adult Orang Asli (Aborigines) from different locations in Perak.<br><br>A cross-sectional parasitological survey was carried out in 3 areas namely; Pos Poi, Perak; Kampong Kerawat and Pergum, Sungai Pulai, Perak; and Kampong Pelantok, Sungai Siput, Perak. Only those who volunteered in the three areas were recruited for the study that was conducted in 2011. Soil samples was collected from the top 2-4 cm layer of the ground using a spoon from several vicinity around the houses especially near the toilet, under the houses and places where the children play. The soil samples were stored in the laboratory refrigerator and examined using the brine floatation method.	Of the 77 respondents, 39 (50.6%) were positive for at least a single parasite. Males had 41.7% infection rates and females with 54.7% infection rates. The most common parasite detected was *Trichuris trichiura* (39%) followed by *Ascaris lumbricoides* (29.2%), *Giardia lamblia* (5.2%), *Entamoeba coli* (5.2%), *Blastocystis* (5.2%), and hookworm (3.9%) infection. Single infections (24.7%) were more common than double (18.2%), triple (6.5%) and quadruple (1.3%) infection.	The current study found only 1.3% infection with *E. histolytica* and 5.2% with *G. lamblia Blastocystis* (5.2%) infections. The decrease in the number of cases of intestinal protozoan infection in this study suggests that clean water supply to the Orang Asli communities has helped in decreasing the number of cases of water borne infections.	5
11	Gee & Kamel, 2020 [48]	116 schoolchildren aged between 6 and 12 years Aboriginal schoolchildren at RPS Banun, Gerik, and Perak, Malaysia.	Institute for Medical Research, Kuala Lumpur	Objective: To identify prevalence of gut parasites among schoolchildren<br><br>Stool samples were collected from 116 schoolchildren aged between 6 and 12 years old; 56 were boys and 60 girls. Fecal samples were examined by direct wet mount, trichrome staining and formalin-ether sedimentation	Overall, 65.5% (76/116) of the schoolchildren were found to be infected with at least one intestinal protozoan species. Of these, 51.3% (39/76) had multiple intestinal protozoan infections with the majority participants harboring two protozoan species concurrently (69.2%, 27/39). The overall prevalence of *Entamoeba histolytica*, Entamoeba coli, Giardia lamblia, *Chilomastix mesnili* and *Blastocytis hominis* infections were 46.6% (54/116), 34.5% (40/116), 21.6% (25/116), 5.2% (6/116) and 1.7% (2/116), respectively. The prevalence of these infections did not show significant age- or gender-dependency relationship, even though higher rates were observed among those aged ≤ 9 years or boys. The co-infection of E. *histolytica* with E. *coli* was most prevalent (46.2%; 18/39) among the schoolchildren, followed by triple infection with E. *coli*, E. *histolytica* and G. lamblia (23.1%; 9/39), and co-infection with E. *histolytica* and G. *lamblia* (10.3%; 4/39).	This study highlighted that single or multiple intestinal protozoan infections are prevalent among schoolchildren in RPS Banun. Therefore, effective and sustainable control measures should be implemented including health education and periodic chemotherapy to reduce the prevalence of protozoan infections in aboriginal community.	5
12	Hakim et al., 2007 [49]	Orang Asli (aborigine) in the Cameron Highlands, Pahang State, and Peninsular Malaysia, where rotavirus was later implicated as the cause.	Institute for Medical Research, Kuala Lumpur	Objective: to investigate the outbreak of acute diarrheal illness occurred among the Orang Asli (aborigine) in the Cameron Highlands<br>Stool specimens were collected from patients admitted to the Cameron Highlands hospital as well as from symptomatic individuals from the villages in the Resettlement Posts. Stool samples were also collected from asymptomatic individuals, particularly the contacts of symptomatic cases and individuals. The stool samples were collected in sterile, screw-capped containers. In the field (and at the hospital), the stool was divided into 3 portions, a part transferred into a container containing polyvinyl alcohol (PVA) and sodium acetate-acetic acid-formaldehyde (SAF) preservative.	Seven different intestinal parasites were detected in the stool samples. They were the soil transmitted helminthes (STH) namely *Ascaris lumbricoides* (25.7%), *Trichuris Trichiura* (31.1%) and hookworm (8.1%), and intestinal protozoa, which included *Giardia lamblia* (17.6%), *Entamoeba histolytica/E.dispar* (9.4%), *Blastocystis hominis* (8.1%) and *Cryptosporidium parvum* (2.7%). Forty-four (59.5%) were infected with at least one parasite and 24 (32.4%), 12 (16.2%) and 8 (10.8%) had single, double and triple parasitic infections, respectively.	This investigation reflects the difficulties and challenges faced by health program managers in addressing intestinal parasitosis among aborigines. Provision of good housing, safe water supply and latrine facilities, as was the case in this particular community, are not good enough if the communities themselves do not appreciate, understand or choose to ignore the importance of environmental sanitation and personal hygiene and clean practices in the prevention of these diseases.	4
13	Elyana et al., 2016 [50]	Two Orang Asli communities in Terengganu comprised of two villages, Sungai Pergam village in Kemaman District and Sungai Berua village in Hulu Terengganu District.	Department of Parasitology, University of Malaya	Objective: to investigate and compare the prevalence, associated risk factors and people's knowledge, attitude and practices (KAP) towards IPIs among the Orang Asli and the Malay populations in rural Terengganu, Malaysia.<br><br>Questionnaire survey was used. Direct smear (wet mount) was applied on all samples and then formalin-ether sedimentation technique was used to increase the detection rates especially when the parasites are in few numbers. Kato Katz technique was used for egg counts to estimate the intensity of infections and the results were recorded as eggs per gram of stool (EPG) Faecal smears were prepared andstained with modified Ziehl-Neelsen stain.	149 (90.3 %) Orang Asli and 43 (24.6 %) Malay children were infected by at least one parasite species. The overall prevalences of intestinal polyparasitism among the Orang Asli and Malay were 68.5 % (113/165) and 14.3 % (25/175), respectively. Multiple logistic regression analysis showed that using unsafe water supply as a source for drinking water, the presence of domestic animals, not wearing shoes when outside, not washing vegetables before consumption, not washing hands after playing with soil, indiscriminate defecation and the low level of mother's education were the key risk factors for intestinal polyparasitism among the Orang Asli, while working mothers and the presence of domestic animals were the risk factors among the Malay children. Almost all the Malays were well aware about the IPIs while Orang Asli respondents had a poor level of related awareness.	This study demonstrates that IPIs are highly prevalent in rural Terengganu, Malaysia. Community awareness about IPIs was found to be imperative in protecting Malay children from these infections. An integrated control programme for the prevention and control of Intestinal parasitic infections is highly recommended for these communities, with a special emphasis on the Orang Asli population.	6
14	Abdulsalam et al., 2012 [51]	300 primary schoolchildren (150 males and 150 females) in Lipis and Raub, Pahang	Department of Parasitology, University of Malaya	Objective: to identify prevalence of gut parasites among schoolchildren<br><br>Direct smear microscopy (detection of *Blastocystis*) and Questionnaire on demographic and socio-economic data, environmental factors, and history of GIT symptoms were used. Statistical analysis SPSS v.13; Chi-square test (associations between independent variables and *Blastocystis* infection); Multiple logistic regression model (identify significant predictors of infection); Approved by the Medical Ethics Committee UMMC.	The overall prevalence of *Blastocystis* infection was 25.7% (77/300) and higher in children with gastrointestinal symptoms (34.7% compared to 2.7%). More frequent in males than females, but not statistically significant. Prevalence was higher among aboriginal children than the Malay but not statistically significant (29.3% compared to 20.2%). About half (49.4%; 38/77) of *Blastocystis* infection were single infection and only 2.3% had both *Blastocystis* and *G. duodenalis*, while 5.3% had *Blastocystis* with *Ascaris* and/or *Trichuris*. Other parasites detected: Trichuris (47%), Ascaris (20.7%), hookworm (1.7%), G. *duodenalis* (15.3%), E. *histolytica/dispar* (4.3%). Significant predictors of *Blastocystis* infection are absence of piped water supply and low level of mother's education.	*Blastocystis* infection exists among primary schoolchildren. Water supply and maternal education were the significant predictors of *Blastocystis*. Need improvement of sanitary facilities and quality of drinking water, public education, and health promotion.	5
15	Sinniah & Rajeswari, 1994 [52]	729 children whose ages ranged from 1-13 years. (391 boys and 338 girls). Single stool specimens were collected from two oil palm and rubber estates situated in Selangor, Malaysia. Stools examined consisted of 614 samples obtained from surveys of parasitic infections among children who come from the rural areas of Selangor, Malaysia and 115 pediatric patients who were admitted for diarrhea.	Department of Parasitology and Department of Social and Preventive Medicine\ Faculty of Medicine, University of Malaya, Kuala Lumpur, Malaysia	Objective: To determine epidemiology *Blastocystis hominis* infection among children and its possible role in causing diarrhea.<br><br>Single stool specimens were collected. The plastic bags for collection of stool was distributed one day prior to the actual collection of feces. Samples returned to the laboratory and stored in a cold room (at 4°C) were all examined within a week. Fecal examinations were carried initially using the direct and formal ether sedimentation techniques to detect protozoan cysts. Semi diarrheic and watery stools were also stained by using the modified Ziehl-Neelsen technique, but most were examined using iodine-stained preparation.	Of the 729 stool samples collected, 18.1% of them were found to be positive for one or more intestinal protozoan parasite. The most common protozoan parasite detected was *G. intestinalis* (8.4%) followed by *E. coli* (7.1 %) and *E. histolytica* (5.1 %). *Blastocystis hominis* (1.1%) was the least common parasite detected. The parasites were more common among the 1-6 year age groups (22.4%) a least among the older children (11.8%). There was no significant difference between the genders. Of the 113 diarrhea cases, 27.4% were found to be positive for intestinal protozoan parasites. The most common protozoan detected was *G. intestinalis* (20.4%) followed by *E. coli* (15.9%) and *E. histolytica* (9.7%). *Blastocystis* was detected in 4.4% of the chidren who were admitted to the pediatric ward of the Univversity Hospital, Kuala Lumpur. The overall prevalence rate for *Blastocystics* among normal and diarrheic children was only 1.1%. Of the eight cases of *Blastocystis* infection reported in this study, in five of them this parasite existed alone by itself without any other parasitic infections. The stool did not show any evidence of bacterial or fungal infection. The five cases were associated with diarrhea but the possibility of viral infection or allergic reaction cannot be dismissed.	The pathogenicity of *B. hominis* should not be ruled out because it is possible that there are some strains of *B. hominis* which may be pathogenic and this may explain why some researchers have reported pathogenic conditions. Infection with *B. hominis* occurs mainly through fecal pollution and as such, it is a public health problem. Patients with a history of diarrhea should be investigated for *Blastocystis* as one of the pathogens during their investigation in the absence of other organisms.	6

16	Abd Ghani & Alharazi, 2016 [53]	Cross-sectional study<br>Sample size: 307<br>Age: 6-14 years<br>Schoolchildren (Orang Asli)<br>(156 Males and 151 females	Department of Medical Microbiology, Universiti Sains Malaysia	Objective: To identify prevalence of *Blastocystis* among school children<br><br>Direct microscope of stool specimens	The prevalence of *Blastocystis* was 21.2%. And associated factors: Females and children aged 12-14 years had a significantly higher prevalence.	Blastocystosis still represents a serious public health problem in aboriginal communities, especially among children.	3
17	Kamel & Najah, 2021 [54]	208 Orang Asli primary schoolchildren of SK Senderut, Pahang from standard 1-6	Department of Parasitoloy, Faculty of Medicine, Universiti Kebangsaan Malaysia	Objective: to identify prevalence of *Blastocystis* among Orang Asli schoolchildren<br>Stool samples were examined using two techniques: Direct Fecal Smear (DFS) & Formalin-ether concentration techniques (FEC);informed consent obtained; no ethical approval certificate mentioned; no statistical analysis mentioned in methodology section	A total of 68 students (32.7%) were found to be positive with *Blastocystis*: Gender (not significant); 38 (33.9%) of female were infected; 30 (31.3%) of male were infected. Age Group (not significant); 40 (34.8%) of upper primary school were infected 93 (30.1%) of lower primary school were infected. Test Method; FEC had a better detection - 62 (88.2%) compared to DFS - 40 (58.8%).	The high prevalence of blastocystosis indicates that it is still a significance health issue in Malaysia. Still necessary to promote health awareness and good hygiene practices especially in school children	3
18	Al-Delaimy, 2014 [55]	783 schoolchildren from 2 primary schools, Kuala Koyan & Pos Betau in Kuala Lipis, Pahang.	Department of Parasitology, University of Malaya	Objective: To determine the prevalence of polyparasitism & associated risk factors by comparing children with multiple infections with those of single or not infected.<br><br>Fecal samples were examined by using direct smear, formalin-ether sedimentation, trichrome stain, modified Ziehl Neelsen stain, Kato-Katz, and Harada Mori techniques. Pretested questionnaire captured Demographic, socioeconomic, environmental, and personal hygiene information. Pearson's x2 test used to investigate association between polyparasitism and demographic, socioeconomic and personal hygiene information. Factors as explanatory variables.	Examination of fecal sample found to be positive for *Blastocystis* sp. (15.1%). 98.4% of the children were found to be infected by at least one parasite species. Of these, 71.4% had polyparasitism; *T. trichiura* and *A. lumbricoides* coinfection was the highest (54.0%). Significant risk factors associated with intestinal polyparasitism are drinking unsafe water, presence of other family members infected with IPI, not washing vegetables before consumption, absence of a toilet in the house, not wearing shoes when outside, not cutting nails periodically, and not washing hands before eating.	Polyparasitism is very common among Orang Asli school children in rural Malaysia. High prevalence of *Blastocystis* sp. And other intestinal commensals were also reported.	7
19	Adli & Kamel, 2020 [56]	92 Orang Asli school children aged 7-12 years at SKTAR, Kuala Lubu Bharu	Department of Parasitoloy, Faculty of Medicine, Universiti Kebangsaan Malaysia	Objective: to identify prevalence of *Blastocystis* among Orang Asli schoolchildren.<br><br>Stool samples were examined using two techniques; Direct Fecal Smear (DFS) & Formalin-ether concentration techniques (FEC) and informed consent obtained.	Very high prevalence of total *Blastocystis* (83.7%) reported. Infection was higher in females (86%) compared to in males (81.6%). Slightly higher in lower primary (7-9 yo) 85.1% compared to higher primary (10-12 yo) 82.2%. FEC had a better detection (66.2%) compared to DFS (51.9%).	These children are still exposed to the many risk factors that influence the spread of protozoa via fecal-oral route. It is necessary to promote health awareness and good hygiene practices especially in school children.	3
20	Salim et al., 1999 [57]	A total of 105 stool samples were collected from animal handlers from 2 local research institutions, a local zoo, and a local abattoir and 163 stool samples were collected from normal healthy individuals	Department of Parasitology, Faculty of Medicine, University of Malaya, Kuala Lumpur, Malaysia	Objective: To identify prevalence of parasites among animal handlers<br><br>The *in vitro* culture method, direct faecal examination and the formalin-ether concentration technique were carried out on each sample to be assessed for fecal pathogens. Stool smears on glass slides were stained with trichrome, modified trichrome, and Ziehl-Neelsen stain for *Entamoeba histolytica*, Microsporidium, and Cryptosporidium, respectively. Stools were also cultured for *Blastocystis* in bijou bottles containing 3 ml of Jones medium for 24 h at 37 °C, and the contents were examined using light microscopy.	The *in vitro* culture method used in the study detected that 41% of 105 animal handlers and 17% of 163 at-dwellers in the city were positive for *Blastocystis*. This statistically significant finding shows that people who work closely with animals do stand at risk of acquiring *Blastocystis* infection.	The preliminary finding shows that people who come into close contact with animals have a greater chance of being infected with *Blastocystis.*	3
21	Suresh et al., 2001 [58]	A total of 187 stool samples were collected randomly in 1998 from the 4 main blocks of flats in Jalan San Peng, Kuala Lumpur.	Department of Parasitology, Faculty of Medicine, University of Malaya, Kuala Lumpur, Malaysia	Objective: To report on the prevalence of B. *hominis* in asymptomatic individuals.<br><br>Stool samples were collected in stool cups, provided on the day prior to collection, and were analysed for the presence of ova, cysts and parasites using standard direct faecal examination and formalin-ether concentration techniques.<br><br>Stool smears on glass microscope slides were stained with Trichrome, modified Trichrome and modified Ziehl-Neelsen stains for *Entamoeba histolytica*/dispar complex trophozoites, microsporidian spores and *Cryptosporidium* oocysts, respectively.	*Blastocystis* was present in 14.9% of stools of flat dwellers, although none admitted any clinical signs or symptoms. *B. hominis* was most prevalent in the 4 l-50 years age-group, with no significant difference in distribution between males and females, or flat dwellers residing in the different blocks of flats. Also present were *Giardia lamblia* (2.14%), *Entamoeba coli* (053%), *Clonorchis sinensis* (2.14%), *Ascaris lumbricoides* (1.07%), *Trichuris trichiura* (2.40%) and *Enterobius vermicularis* (0.53%). Thus, 27.9% of the population surveyed harboured one or more of these parasites.	The findings confirm that *B. hominis* can be found frequently in indigenous, asymptomatic city dwellers, despite the improvement in sanitation and life-style due to urbanization.	5
22	Menon et al., 1999 [59]	Prospective study<br>Sample size: 237<br>Age: 9 months-11 years (those with cancer; leukaemia and solid tumour)	Hospital Sains Malaysia, Kota Baru	Objective: To identify prevalence of *Blastocystis* among pediatric patients<br>Direct microscope of stool specimens	The prevalence of *Blastocystis* was 4%.	There is high prevalence of enteric parasite among children of cancer. Helminths were more common than protozoa. Most of children were asymptomatic	3
23	Thergarajan et al., 2019 [60]	A hospitalized-based cross-sectional study was conducted on patients who were admitted at UMMC due to dengue fever.	Department of Parasitology, Department of Medicine, University of Malaya	Objective: To determine the association between *Blastocystis* prevalence and severity of gastrointestinal symptoms in dengue patients and to determine the association between body temperature and *Blastocystis* proliferation.<br><br>Stool samples were collected from patients suffering from dengue fever. Each patient was provided with stool cup and sample was processed immediately after collection.<br><br>Faecal examination was performed by inoculating approximately 50mg of stool sample into 3ml of Jones' medium supplemented with 10% horse serum. It was incubated at 37˚C and examined using light microscope for the subsequent three days. A total of 89 stool samples were collected and cultures were continuously examined at 24, 48, and 72 hours.	Continuous culture examination showed that 21 samples were positive, revealing a distribution of 23.6% *Blastocystis* sp. infection among dengue patients. The genomic DNA of these 21 positive samples were harvested and further subtyped. Sequence analysis categorized positive samples into four subtypes, subtype 1 (7 = 33.33%); subtype 3 (10 = 47.62%); subtype 4 (3 = 14.29%), and subtype 6 (1 = 4.76%).	Firstly, the severity of dengue symptoms should be correlated with presence of *Blastocystis* sp. infection.<br><br>Second, it is also important to make correlation between different age groups, as this can influence the susceptibility towards dengue and *Blastocystis* sp. infection in both adult and pediatric population.	5
24	Tan et al., 2009 [61]	A total of 311 cancer patients and 247 HIV-infected individuals who attended to local hospitals were examined for *Blastocystis* infection.	Department of Parasitology, Faculty of Medicine, University of Malaya, Kuala Lumpur, Malaysia	Objective: To determine the prevalence of *Blastocystis sp.* in both cancer and HIV/AIDS patients<br><br>The parasites were isolated from patients' fecal sample by in vitro cultivation at 37°C using Jones' medium (Jones 1946) supplemented with 10% horse serum (Tan et al. 2008). Twenty *Blastocystis* sp. isolates were randomly selected from each group for genotyping analysis. PCR was performed using seven pairs of STS primers.	Ten (9.7%) of the pre-treatment groups were positive with *Blastocystis sp.* while 14 (6.7%) were positive in the post-treatment group. A total of 24 (7.7%) of the cancer patients were found to be infected with the parasite. Meanwhile, a total of 49 (19.8%) of the 247 HIV/AIDS patients were coinfected with *Blastocystis* sp. The results for genotyping of the 40 isolates of *Blastocystis* sp. showed that *Blastocystis* subtypes 1, 2, 3, and 4 were found. None of the isolates was classified as subtype 5–7. Subtype 3 was the most prevalent followed by subtypes 4, 1, and 2. Two isolates obtained from HIV/AIDS patients showed negative PCR amplification with all seven sets of STS primers and were probably unknown subtypes.	The present study highlights that *Blastocystis* sp. is not rare and should be looked for routinely in immunocompromised patients who have gastrointestinal complaints.<br><br>Since *Blastocystis* sp. isolates obtained in the present study fall into the potentially pathogenic genotypes, treatment should be considered if the symptoms persist.	6
25	Sahimin et al., 2020 [62]	Migrant workers<br>(Sample size: 220) living in high numbers in major cities	Department of Parasitology, Faculty of Medicine, University of Malaya, Kuala Lumpur, Malaysia	Objective: To identify prevalence of *Blastocystis* among migrant workers living in high numbers in major cities<br><br>Detection of *Blastocystis* was done using the *in vitro* cultivation method and positive samples were subsequently maintained by sub culturing. However, well-grown isolates will be selected for PCR amplification Genomic DNA.	The total prevalence was 30.9%; n = (68/220). ST3 (54.5%; n = 12), followed by ST1 (36.4%; n = 8) and ST2 (9.1%; n = 2). None of the socio-demographic risk factors evaluated were significant.	This study warrants continuous monitoring as well as understanding the impact of transmission among the migrant community with the local population especially those involved in food service sector.	3
26	Angal et al., 2015 [63]	The target population are the prison inmates who are either HIV positive or HIV negative regardless of their race and nationality. This study was carried out at the Kajang Prison, Selangor, Malaysia from June 2012 until January 2013 involving adult male inmates ranging from 21 to 70-years-old.	Department of Parasitology, Faculty of Medicine, University of Malaya, Kuala Lumpur, Malaysia	Objective: to establish firm data on the prevalence and diversity of IPIs (Intestinal Parasitic Infections) among HIV-infected and non-HIV-infected individuals in a prison Questionnaires and samples collection<br>Filled stool containers were collected and blood-drawing session was carried-out. Reserved faecal samples were processed by formalin ether concentration technique followed by iodine staining and were examined via microscopy (100× and 400× magnification) for the presence of intestinal protozoa and helminths.	A total of 294 stool and blood samples each were successfully collected, involving 131 HIV positive and 163 HIV negative adult male inmates whose age ranged from 21 to 69-years-old. Overall prevalence showed 26.5 % was positive for various IPIs. The IPIs detected included *Blastocystis* sp., *Strongyloides stercoralis*, *Entamoeba* spp., *Cryptosporidium* spp., *Giardia* spp., and *Trichuris trichiura*. Comparatively,the rate of IPIs was slightly higher among the HIV positive inmates (27.5 %) than HIV negative inmates (25.8 %). Seropositivity for S. *stercoralis* was more predominant in HIV negative inmates (10.4 %) compared to HIV infected inmates (6.9	Overall prevalence of IPIs among inmates was 26.5 % (78 out of 294). The information gathered from the study will enable the health care providers and prison management staff to understand the trend and epidemiological situations of parasitic coinfections in a prison setup. It also provides evidence based guidance to improve prevention and control (i.e. food preparation, boiled drinking water) through health education as well as treatment management strategies of IPIs co-infections (i.e. parasite infections screening, anti-parasitic treatments)	6

				For detection and confirmation of *Cryptosporidium* spp., Isospora belli and *Cyclospora cayetanensis* oocysts, modified Ziehl-Neelsen staining was performed. Serological test of strongyloidiasis. Molecular characterization of microscopy-positive samples and seropositive samples	%), however these findings were not statistically significant. PCR confirmed the presence of *Blastocystis*, *Strongyloides*, *Entamoeba histolytica* and *E. dispar*.		
